# Deep Sequencing of H7N9 Influenza A Viruses from 16 Infected Patients from 2013 to 2015 in Shanghai Reveals Genetic Diversity and Antigenic Drift

**DOI:** 10.1128/mSphereDirect.00462-18

**Published:** 2018-09-19

**Authors:** Yong-Li Xiao, Lili Ren, Xi Zhang, Li Qi, John C. Kash, Yan Xiao, Fan Wu, Jianwei Wang, Jeffery K. Taubenberger

**Affiliations:** a Viral Pathogenesis and Evolution Section, Laboratory of Infectious Diseases, National Institute of Allergy and Infectious Diseases, National Institutes of Health, Bethesda, Maryland, USA; b MOH Key Laboratory of Systems Biology of Pathogens and Christophe Mérieux Laboratory, Institute of Pathogen Biology, Chinese Academy of Medical Sciences, Beijing, People’s Republic of China; c Shanghai Municipal Center for Disease Control and Prevention, Shanghai, People’s Republic of China; University of Michigan—Ann Arbor; New York University; Tufts University

**Keywords:** DNA sequencing, H7N9, avian viruses

## Abstract

Influenza A virus (IAV) infections are a major public health concern, including annual epidemics, epizootic outbreaks, and pandemics. A significant IAV epizootic outbreak was the H7N9 avian influenza A outbreak in China, which was first detected in 2013 and which has spread over 5 waves from 2013 to 2017, causing human infections in many different Chinese provinces. Here, RNA from primary clinical throat swab samples from 20 H7N9-infected local patients with different clinical outcomes, who were admitted and treated at one hospital in Shanghai, China, from April 2013 to April 2015, was analyzed. Whole-transcriptome amplification, with positive enrichment of IAV RNA, was performed, all 20 samples were subjected to deep sequencing, and data from 16 samples were analyzed in detail. Many single-nucleotide polymorphisms, including ones not previously reported, and many nonsynonymous changes that could affect hemagglutinin head and stalk antibody binding epitopes were observed. Minor populations representing viral quasispecies, including nonsynonymous hemagglutinin changes shared by antigenically variant H7N9 clades identified in the most recent wave of H7N9 infections in 2016 to 2017, were also identified.

**IMPORTANCE** H7N9 subtype avian influenza viruses caused infections in over 1,400 humans from 2013 to 2017 and resulted in almost 600 deaths. It is important to understand how avian influenza viruses infect and cause disease in humans and to assess their potential for efficient person-to-person transmission. In this study, we used deep sequencing of primary clinical material to assess the evolution and potential for human adaptation of H7N9 influenza viruses.

## Abstract (precis)

H7N9 subtype avian influenza viruses caused infections in over 1,400 humans from 2013 to 2017 and resulted in almost 600 deaths. It is important to understand how avian influenza viruses infect and cause disease in humans and to assess their potential for efficient person-to-person transmission. In this study, we used deep sequencing of primary clinical material to assess the evolution and potential for human adaptation of H7N9 influenza viruses.

## INTRODUCTION

Influenza A virus (IAV) infections are a major public health concern, including annual epidemics, epizootic outbreaks, and occasional pandemics (1). Historically, there have been at least 14 influenza pandemics in the last 500 years (2) and 4 in the last 100 years, including the “Spanish” influenza A/H1N1 pandemic in 1918 that resulted in at least 50 million deaths worldwide (3) and the most recent one, in 2009, caused by a novel swine-origin influenza A/H1N1 strain which may have resulted in up to 284,000 deaths globally (4).

Influenza epizootic outbreaks, in which animal-derived IAV infections cause localized outbreaks in the absence of sustained human-to-human transmission, can cause high levels of morbidity and mortality. Such epizootic outbreaks may also presage the emergence of a stably human-adapted IAV strain capable of causing a pandemic. One of the most significant IAV epizootic outbreaks of the past decade is the H7N9 avian influenza A outbreak, first detected in China in March 2013 (5). H7N9 human spillover infections have spread over 5 waves to many different Chinese provinces, and a number of imported H7N9 cases have been reported in other countries (6, 7). As of 7 December 2017, a total of 1,566 laboratory-confirmed cases of human infection with avian influenza A/H7N9 viruses, including at least 613 deaths, have been reported, with a case fatality rate (CFR) of approximately 39% (8). Previously published studies examined the origin, spread, and continued genetic and antigenic evolution of H7N9 viruses since the emergence of the strain in 2013, including frequent reassortment with other avian IAVs, particularly H9N2 viruses (9, 10); acquisition of mutations associated with mammalian adaptation (7, 8, 11); and reduced susceptibility to viral neuraminidase (NA) inhibitor-based antiviral drugs (6).

During the first 4 waves, or epizootic clusters, of human H7N9 cases (2013 to 2016), almost 90% of diagnosed patients developed pneumonia, approximately two-thirds required intensive care, and 41% had a fatal outcome (6). The H7N9 viral isolates responsible for the most recent (fifth) wave (2016 to 2017) have been shown to have evolved into two distinct genetic lineages, referred to as the Pearl River Delta and Yangtze River Delta lineages, with most recent isolates belonging to the Yangtze River Delta lineage (6, 8). Importantly, viruses in this lineage show reduced antigenic relatedness to candidate prepandemic H7N9 vaccine virus stocks developed in 2013 (6).

Several different methods have been developed to detect IAV infection in patients by the use of different approaches, including methods based on tissue culture, antibody titers in serological assays, nucleic acid amplification, and nucleic acid sequencing (12). Since their commercialization in 2005, next-generation sequencing technologies have evolved dramatically, especially regarding sequencing capacity, which has increased by factors of 100 to 1,000 (13). These sequencing technologies have been widely used in influenza research studies, including detecting IAV and norovirus infections in patients (14), uncovering mixed infection with 2009 pandemic IAV (15), high-throughput sequencing of influenza B viruses (16), evaluating genetic stability of influenza vaccine viruses (17), revealing antigenic variants at low frequencies in IAV-infected patients (18), and high-throughput identification of IAV H3N2 antigenic drift variants (19). However, each of these approaches uses virus-specific primers and virus-specific PCR amplification strategies. Due to the ability of next-generation sequencing to detect low-abundance sequences, avoidance of PCR-introduced errors would be helpful in sequence analyses. It has been shown that most commonly used PCR enzymes, including high-fidelity enzymes, have error rates of 10^−5^ to 10^−6^ point mutations/base pair/duplication (20). Besides well-characterized polymerase base substitution errors, other sources of error have been found to be equally prevalent, including PCR-mediated recombination, template switching, and DNA damage introduced during temperature cycling (21). In addition, lower-quality or partially degraded RNA, particularly that present in clinical or archival samples, can pose a great challenge for PCR-based amplification using influenza virus universal primers (22) or specific primers.

In the current study, we applied a whole-transcriptome amplification (WTA) method, with positive selection of IAV H7N9 viral RNA sequences by target enrichment hybridization, and next-generation sequencing technologies. Using a combined WTA/positive-enrichment target hybridization method, we sought to perform analyses that could be applied to routinely obtained clinical samples that contain limited amounts of RNA, often with degradation. Recently, Briese et al. demonstrated that a similar target enrichment approach with as little as 40% sequence identity of a targeted genome can be used for preparing a comprehensive probe library (23). Using these approaches, we directly sequenced 20 clinical throat swab samples from H7N9-infected patients in Shanghai, China, with different clinical outcomes from 2013 to 2015 by the use of Illumina next-generation sequencing technology. By analyzing sequences from 16 of the 20 samples in detail, we obtained both the consensus viral sequences from each patient with a H7N9 IAV infection and minor populations representing viral quasispecies. Although the samples analyzed in this study were from only one region, Shanghai, China, examination of the viral quasispecies revealed several hemagglutinin nonsynonymous mutations shared with the antigenically variant H7N9 clades identified in the most recent waves of H7N9 infections in 2016 to 2017 (6, 8). In addition, the deep-sequencing technology revealed a number of single-nucleotide polymorphisms (SNPs) in our samples that have not been previously reported in humans.

## RESULTS

### Patient information.

In this study, throat swabs from 20 reverse transcription-PCR (RT-PCR)-confirmed H7N9-infected patients who were hospitalized in Shanghai, China, from 2013 to 2015 were processed. Available clinical information from the patients in the study, including symptom onset, hospitalization and sampling dates, antiviral/antibiotics usage, and patient outcome, is shown in Table 1. Among the cases, 7 were infected during wave 1 (6–8), with hospitalization admissions from 3 April to 16 April 2013. There were 2 deaths in this cohort, with a case fatality rate (CFR) of 28.6%. There were 9 cases during wave 2, with hospitalization admissions from 30 December 2013 to 20 January 2014. There were 3 cases for which outcome information was not available, while the remaining 6 patients all died; thus, for this cohort, the CFR was ≥66.7%. There were 4 cases in wave 3, with hospitalization admissions from 17 January to 3 April 2015. There were 3 deaths in this cohort, with a CFR of 75.0%. Overall, the CFR in these hospitalized H7N9-infected patients was ≥55%.

**TABLE 1:** Summary of all patient data^*a*^

Sample	Date of onset ofsymptoms	Hospital admission date	Sampling date	Outcome	Antivirus therapy	Antivirus therapy date	Antibiotics
1	30 March 2013	3 April 2013	7 April 2013	Discharge	Tamiflu	3 April 2013	Yes
2	9 April 2013	16 April 2013	17 April 2013	Death	Tamiflu	17 April 2013	Yes
3	4 April 2013	8 April 2013	12 April 2013	Discharge	Tamiflu	12 April 2013	Yes
4	25 March 2013	5 April 2013	7 April 2013	Death	Tamiflu	5 April 2013	Yes
5	15 March 2013	12 April 2013	12 April 2013	Discharge	Tamiflu/Ribavirin	12 April 2013	Yes
6	4 April 2013	9 April 2013	10 April 2013	Discharge	Tamiflu	10 April 2013	Yes
7	30 March 2013	4 April 2013	6 April 2013	Discharge	Tamiflu	4 April 2013	Yes
8	26 December 2013	30 December 2013	3 January 2014	Death	Yes	2 January 2014	Yes
9	1 January 2014	8 January 2014	10 January 2014	Death	Yes	9 January 2014	Yes
10	3 January 2014	9 January 2014	16 January 2014	Death	NA	NA	NA
11	NA	NA	NA	NA	NA	NA	NA
12	6 January 2014	20 January 2014	28 January 2014	Death	Yes	20 January 2014	Yes
13	NA	NA	17 January 2014	NA	NA	NA	NA
14	10 January 2014	17 January 2014	18 January 2014	Death	NA	NA	Yes
15	11 January 2014	17 January 2014	18 January 2014	Death	Yes	17 January 2014	Yes
16	NA	NA	30 January 2014	NA	NA	NA	NA
17	3 January 2015	17 January 2015	22 January 2015	Death	Yes	21 January 2015	Yes
18	4 February 2015	20 February 2015	11 February 2015	Death	Yes	10 February 2015	Yes
19	26 March 2015	1 April 2015	3 April 2015	Discharge	Yes	1 April 2015	Yes
20	27 March 2015	3 April 2015	6 April 2015	Death	Yes	5 April 2015	Yes

a NA, not available.

### Sequence analysis of positive- and negative-control experiments.

Prior to experimental sequence analysis of the patient samples, positive- and negative-control experiments were designed and performed to evaluate the WTA/enrichment method. The sequencing library for the positive control was made from RNA that had been reverse transcribed from the hemagglutinin (HA) open reading frame (KC853766.1) from A/Hangzhou/1/2013 (H7N9) cloned into pBluescript II KS(-). The transcribed HA RNA was processed by the use of the same pipeline, including linear amplification, H7N9 probe enrichment, sequencing, and analysis. Because of the high sensitivity of high-throughput sequencing technology, we also performed a negative-control experiment to determine a baseline of influenza virus contamination in the construction of the libraries. For the negative control, a sequencing library was made from HeLa cell total RNA following the positive-control experiment using the same process pipeline.

From the positive-control HA RNA library, we obtained 355,521,326 reads; 342,112,470 (96.23%) of them aligned to the H7N9 HA reference segment. Similarly, after SNP analysis performed without any specific requirements, the highest SNP value in this run was 2.3% at HA nucleotide position 1144 (46622 reads as T; 1114 reads as A), with coverage of 47,766 reads (Table 2). This provided a baseline of the noise in the library construction and sequencing pipeline. It has been reported that the error rate of the Illumina platform generally increases toward the end of the read, reaching as high as 5% (24). Therefore, to reduce the potential false-positive SNP calling rate, we decided to use 10% as the SNP cutoff level for this study. We obtained 292,901,554 total reads from the negative-control experiment, and there were 3,815 (0.0013%) reads aligned to H7N9 consensus sequences. Therefore, we used this number of sequence reads as an approximate baseline for the sequence contamination level for this study (see below).

**TABLE 2:** Base changes from positive control^*a*^

Reference	nt position	Reference base	Coverage (no. of reads)	No. of insertions	No. of deletions	No. (%) of substitutions					Avg *q* value
						A	T	G	C	N	
gi_475662453_HA	79	G	39,651	1	0	A = 25 (0.1)	T = 487 (1.2)	ref (G)	C = 17 (0.0)	N = 0 (0.0)	32.16
gi_475662453_HA	1144	T	47,766	1	2	A = 1,114 (2.3)	ref = T	G = 5 (0.0)	C = 24 (0.1)	N = 0 (0.0)	33.958
gi_475662453_HA	1146	G	47,155	0	0	A = 1,003 (2.1)	T = 52 (0.1)	ref (G)	C = 7 (0.0)	N = 0 (0.0)	33.858

a nt, nucleotide; *q*, false-discovery rate; ref, reference nucleotide.

### H7N9 viral cDNA library sequence analysis.

**FIG 1:**
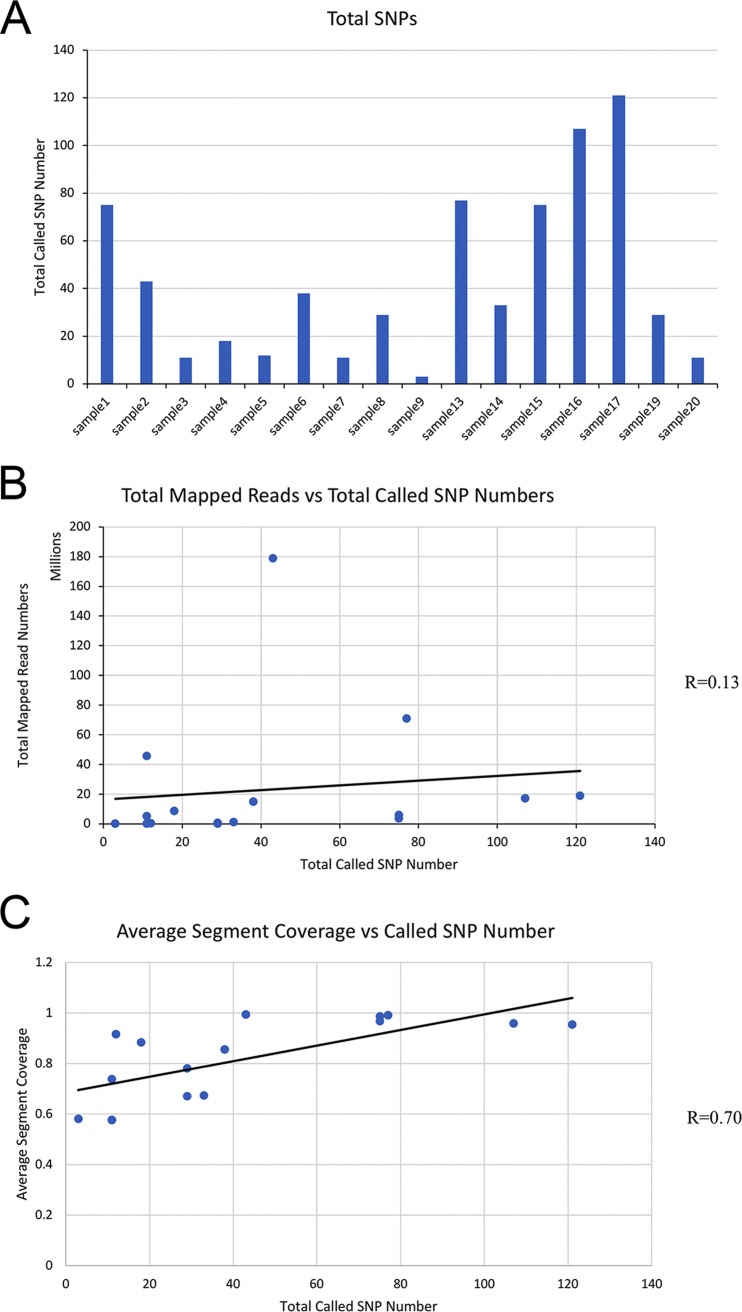
Mapped reads and called SNP numbers. (A) Numbers of called SNPs for all samples. (B) Correlation of the number of mapped reads and the number of called SNPs. (C) Correlation of the average coverage and the number of called SNPs.

Illumina deep sequencing of enriched cDNA libraries was performed on samples from 20 patients that were H7N9 RT-PCR positive. The summary of the sequencing results is shown in Table 3, and data corresponding to the average sequencing depth of each segment of the genome from all the samples are shown in Table 4. Between 42 million and 324 million reads (reflecting the sum of reads produced in both directions) were generated from the 20 patient sample libraries. After mapping to the reference human H7N9 consensus sequence from an isolate collected in China in 2013, the total number of mapped reads ranged from 3,245 reads to over 178 million reads among the patient sample libraries. Because there were 3,815 mapped reads in our negative-control experiment, samples 10 (number of mapped reads, 10,693), 11 (number of mapped reads, 3,534), 12 (number of mapped reads, 3,830), and 18 (number of mapped reads, 3,245) were eliminated from further SNP analysis to avoid potential false-positive results. Therefore, compared to the human 2013 consensus H7N9 sequence, a total of 509 SNPs were detected from these 16 remaining patient sample libraries, with 454 unique SNP positions identified (Fig. 1A). Synonymous and nonsynonymous SNPs that passed the filter criteria utilized (described in Materials and Methods) from each patient are shown in Table 3 (see also Table S1 in the supplemental material).

**TABLE 3:** Summary of all sample data

Sample	Read type	Read length (bp)	Total no. of reads produced/ no. of left reads produced	No. of right reads produced	Total no. of reads mapped to H7N9/no. of left reads mapped	No. of right reads mapped	Coverage (%)								Avg coverage	Total no. of SNPs	No. of nonsynonymous SNPs	Total no. of mapped reads
							2013H7N9_ consensus_ NA	2013H7N9_ consensus_ HA	2013H7N9_ consensus_ M2_M1	2013H7N9_ consensus_ PB2	2013H7N9_ consensus_ PB1	2013H7N9_ consensus_ PA	2013H7N9_ consensus_ NP	2013H7N9_ consensus_ NEP_NS1				
1	Single end	80	1.1E+08		3,637,318		0.9979	1	1	0.9996	0.9213	1	0.9947	0.9869	0.9875	75	48	3,637,318
2	Paired end	100	1.6E+08	1.6E+08	9.4E+07	8.5E+07	0.9785	1	0.9807	1	1	0.9967	1	1	0.9945	43	22	1.8E+08
3	Paired end	100	6.2E+07	6.2E+07	2.6E+07	2E+07	0.4256	0.5264	0.9165	0.5298	0.1033	0.4816	0.9993	0.6313	0.5767	11	8	4.6E+07
4	Paired end	100	3.4E+07	3.4E+07	4,519,318	4,315,814	0.6009	1	1	0.9662	0.6306	0.9805	0.8971	0.9976	0.8841	18	6	8,835,132
5	Paired end	100	3.5E+07	3.5E+07	209,949	187,212	0.6481	0.9721	0.999	0.9982	0.8813	0.9921	0.8938	0.9499	0.9168	12	6	397,161
6	Paired end	100	3.9E+07	3.9E+07	7,884,584	7,067,362	0.6059	0.9447	1	0.9763	0.7735	0.9991	0.8838	0.673	0.857	38	8	1.5E+07
7	Paired end	100	6E+07	6E+07	2,784,497	2,463,756	0.4428	0.5502	0.9562	0.5456	0.1579	0.537	1	0.4236	0.5766	11	9	5,248,253
8	Paired end	100	2.7E+07	2.7E+07	369,432	355,017	0.3569	0.5258	0.9552	0.5228	0.2498	0.9865	0.9993	0.7673	0.6705	29	9	724,449
9	Paired end	100	2.2E+07	2.2E+07	126,599	117,241	0.3534	0.4724	0.7994	0.3702	0.1205	0.953	0.8851	0.6981	0.5815	3	1	243,840
10	Paired end	100	2.1E+07	2.1E+07	5,554	5,139	0.5322	0.6144	0.6568	0.6075	0.3914	0.9963	0.8758	0.6516	0.6658			10,693
11	Paired end	125	7.6E+07	7.6E+07	1,821	1,713	0.877	0.9412	0.8248	0.4654	0.7744	0.9893	0.819	0.5668	0.7822			3,534
12	Paired end	125	8.6E+07	8.6E+07	2,055	1,775	0.9049	0.959	0.6263	0.5061	0.9666	0.9893	0.9866	0.6265	0.8207			3,830
13	Paired end	125	9.7E+07	9.7E+07	3.6E+07	3.5E+07	1	1	1	0.9268	1	1	1	1	0.9909	77	17	7.1E+07
14	Paired end	125	8.9E+07	8.9E+07	653,106	647,614	0.5622	0.8259	0.9735	0.3412	0.2933	0.6815	0.8484	0.8675	0.6741	33	14	1,300,720
15	Paired end	125	8.4E+07	8.4E+07	2,981,673	2,871,180	1	1	0.9674	0.7728	0.9996	1	1	1	0.9674	75	20	5,852,853
16	Paired end	125	1E+08	1E+08	8,765,927	8,345,327	0.9964	1	0.9776	0.7018	1	1	1	1	0.9595	107	35	1.7E+07
17	Paired end	125	8.5E+07	8.5E+07	9,650,732	9,228,316	1	1	0.9776	0.6557	1	1	1	1	0.9542	121	23	1.9E+07
18	Paired end	125	9.9E+07	9.9E+07	2,032	1,213	0.9614	0.9899	0.831	0.5456	0.9974	0.9893	0.9572	0.9678	0.905			3,245
19	Paired end	125	8.8E+07	8.8E+07	128,978	123,197	0.6295	0.899	0.834	0.4158	0.8034	0.9777	0.9232	0.7709	0.7817	29	7	252,175
20	Paired end	125	8.2E+07	8.2E+07	53,551	51,599	0.4664	0.9721	0.6151	0.4535	0.9494	0.8052	0.8904	0.753	0.7381	11	6	105,150

**TABLE 4:** Average sequencing depth of all samples

Consensus segment	Avg sequencing depth (no. of reads) for sample:																			
	1	2	3	4	5	6	7	8	9	10	11	12	13	14	15	16	17	18	19	20
PB2_2013	43,366.63	3,176,994.52	211,699.39	94,110.75	6,312.50	76,066.89	41,121.49	1,849.69	5.13	17.37	20.78	21.16	318,682.62	751.59	22,865.95	137,128.67	123,741.12	11.90	1,562.34	8.39
PB1_2013	4,312.23	956,734.17	4,550.66	52,287.47	1,749.74	51,385.22	7.78	8.09	1,020.84	8.80	7.64	17.54	443,424.91	0.33	25,870.11	92,391.27	122,526.39	16.38	64.04	9.62
PA_2013	6,286.60	1,409,587.03	79.50	3,373.22	4,201.35	29,885.45	97,808.97	1,942.12	5.02	27.80	22.78	15.57	747,723.04	20,806.25	102,977.92	74,182.53	117,628.43	33.59	2,052.82	160.04
NP_2013	8,352.22	828,919.44	1,109,193.05	74,219.91	5,392.38	71,960.99	96,481.43	7,431.13	17.97	175.48	105.13	31.73	947,654.98	14,806.53	44,093.52	220,890.95	175,262.27	37.16	10,432.36	18.79
NS_2013	4,221.86	364,374.34	1,279.36	1,733.17	365.75	11.34	0.84	5,633.65	16.83	12.65	2.68	2.54	269,508.95	10,758.95	39,148.42	17,559.82	55,246.42	12.08	1.79	1.49
MP_2013	5,747.54	1,279,071.59	2,493,897.05	23,211.51	1,385.23	838,214.05	1,970.88	3,199.19	8.04	52.85	24.44	14.89	393,944.12	4,970.60	34,398.93	63,874.87	114,203.92	12.60	1,910.13	80.45
HA_2013	24,865.55	985,219.43	8,491.03	238,632.09	1,470.89	57,589.48	35,977.54	25,961.20	13,059.48	369.46	62.83	124.78	1,809,859.77	35,499.85	131,745.77	537,585.12	541,936.27	75.83	2,794.49	7,102.20
NA_2013	43,580.60	817,996.07	4,326.90	3,945.42	63.52	85,819.60	11,151.14	1,638.34	4.36	4.08	2.59	5.72	187,019.31	4,614.81	19,543.10	24,959.23	45,620.17	7.15	21.91	2.24

10.1128/mSphereDirect.00462-18.3 TABLE S1 Synonymous and nonsynonymous SNPs that passed the filter criteria utilized from each patient. Download Table S1, XLSX file, 0.03 MB. Copyright © 2018 Xiao et al. 2018 Xiao et al. This content is distributed under the terms of the Creative Commons Attribution 4.0 International license.

### Comparison of SNPs detected in this study and SNPs detected from previously sequenced H7N9 human isolates.

The precomputed SNP data from human H7N9 IAVs were downloaded on 6 March 2017 from the Influenza Research Database (IRD) (25) with different SNP scores, which were generated from the modified formula as described by Crooks et al. (26). Briefly, the scores represent the normalized entropy of the observed allele distribution at each position. The least polymorphic site would have a score of 0 (no polymorphism), and the most polymorphic site would have a score of 200 (highest polymorphism). Compared to the SNPs from the IRD with a score of >1, among the 454 SNPs identified in the current study, there were 276 previously reported in the IRD. At the level of an SNP score of >10, only 195 SNPs were reported by IRD (Table S1). In addition, at the level of an IRD SNP score of >1, among the same 276 SNP positions, 255 SNP calls were the same as those observed in the current study. There were 178 SNP positions with 106 nonsynonymous changes in human H7N9 that have not been reported at the IRD.

### Comparison of H7N9 viral genome SNPs between different patients.

Among the 16 remaining sequenced and analyzed patient samples, a total of 509 SNPs that passed filter criteria were detected. The number of SNP locations observed in each sample was quite variable, ranging from 3 SNPs in sample 9 to 121 in sample 17 (Table 3). The correlation (*R*) between the number of mapped reads and the called SNP number was low (*R* = 0.13) (Fig. 1A and B). For example, sample 3 had more than 45 million mapped reads but only 11 SNPs. On the other hand, the actual numbers of reads containing the SNPs at the SNP positions were better correlated to the depth at those positions (average *R* = 0.91) (Table S2). When the coverage of the H7N9 genome was examined, the correlation (*R*) of numbers of identified SNP locations in samples and average genome coverages was 0.70 (Fig. 1C). Table 3 suggests that the differences between samples in the numbers of called SNPs were not primarily due to differences in read depth or coverage between samples. Comparing H7N9 sequences from the 16 patients to the reference genome of the consensus sequence of all human H7N9 strains in China in 2013, 509 SNPs, representing 454 unique nucleotide positions, were found. Among them, there were 120 SNP positions shared in two or more samples. Selected amino acid substitutions with known or suspected functional significance with respect to the IAV H7N9 strains observed during the epizootic waves in China (7, 8, 11) from the 16 patient samples analyzed here are listed in Table 5.

**TABLE 5:** Selected amino acid substitutions observed in H7N9 case samples^*a*^

H7N9 protein^*b*^	Codon substitution^*c*^	Amino acid substitution						Function/region	Case no.
		Avian H7N9 consensus 2013	Avian H7N9 consensus 2014	Avian H7N9 consensus 2015	Human H7N9 consensus 2013	Human H7N9 consensus 2014	Human H7N9 consensus 2015		
HA	V104I	V	V	V	V	V	V	HA head	16
	S136N	S	S	N	S	S	N	HA head	17
	A143V	A	A	V	A	A	V	HA head	17
	R148K	R	R	R	R	R	R	HA head	17
	L186I	L	L	I	L	L	I	HA receptor binding	17
	Q235L	Q	L	L	L	L	L	HA receptor binding	1, 2, 4, 13, 15, 16, 17
	R270K	R	R	R	R	R	R	HA head	15, 16
	L394I	L	L	L	L	L	L	HA stalk	8, 13, 14, 15, 16
	E396A	E	E	A	E	E	A	HA stalk	17, 19, 20
	S499R	S	S	R	S	S	R	HA stalk	17, 19, 20
	N551S	N	N	N	N	N	N	HA cytoplasmic tail	1, 2, 3, 6, 8, 13, 14, 15, 16, 20
	G552R	G	G	G	G	G	G	HA cytoplasmic tail	1, 2, 3, 6, 8, 13, 14, 15, 16, 20
									
NA	I16T	I	I	I	I	I	T	NA transmembrane domain	15, 16, 17
	T65A	T	T	T	T	T	T	NA stalk	17
	R148K	R	K	R	R	R	R	NA catalytic site	1
	S242P	S	S	P	S	S	P	NA head	17
	E263D	E	E	E	E	E	E	NA head	15, 16
	R289K	R	R	R	R	R	R	NA catalytic site	16
	N322S	N	N	S	N	N	S	NA head	17
									
PB2	M473V	M	M	M	M	M	M	Nuclear localization signal	15, 16
	M535L	M	L	L	M	M	L	T cell epitope	6, 13, 16, 17
	N559T	T	T	T	N	T	T	T cell epitope	17
	E627K	E	E	E	K	K	K	C-terminal region; pathogenicity determinant	1
	N759K	N	N	N	N	N	N	C-terminal region	1, 2
									
PB1	K237R	K	K	K	K	K	K	T cell epitope	9
									
PB1-F2	No changes								
									
PA	D67Y	D	D	D	D	D	D	T cell epitope	13
	I94T, I94V	I	I	I	I	I	I	N-terminal domain	8: 94T, 7: 94V
	Q556R	Q	Q	Q	Q	Q	Q	PB1-binding region, T cell epitope	6
									
PA-X	D67Y	D	D	D	D	D	D	N-terminal domain	13
	I94T, I94V	I	I	I	I	I	I	N-terminal domain	8: 94T, 7: 94V
	L194P	L	P	P	L	P	P	C-terminal domain	13, 15, 16, 17
	R248K	R	K	R	R	R	R	C-terminal domain	16, 17
									
NP	R348K	R	R	R	R	R	R	NP oligomerization domain	19
	L466F	L	L	L	L	L	L	PB2 binding domain	14, 16
									
M1	N82T	N	N	N	N	N	N	Membrane binding domain	14, 16
	F109L	F	F	F	F	F	F	Membrane binding domain	4, 5, 6
	D156E	D	D	D	D	D	D	Membrane binding domain	3, 7
									
M2	P10L	P	L	L	P	P	L	Ectodomain	2, 13, 15
									
NS1	R67Q	R	R	R	R	R	R	RNA binding domain	14, 16
	S114P	S	S	S	S	S	S	Effector domain	8, 13, 14, 16
	P213S	P	P	P	P	P	P	C-terminal protein interaction domain	14, 16
									
NEP/NS2	No changes								

a Consensus sequences were constructed from all full-length open reading frames available from avian and human H7N9 sequences from 2013, 2014, and 2015 downloaded from the NCBI Influenza Virus Database (https://www.ncbi.nlm.nih.gov/genomes/FLU/Database/nph-select.cgi?go=database).

b H7N9 proteins, including hemagglutinin, are numbered from the initial methionine as codon 1.

c Nonsynonymous mutations observed according to the the H7N9 case criteria stated in Materials and Methods compared to the reference sequence (the 2013 human H7N9 consensus sequence), except for HA codon 235, where changes from the avian consensus are noted.

10.1128/mSphereDirect.00462-18.4 TABLE S2 Actual numbers of reads containing SNPs at the identified SNP positions were better correlated to the depth at these positions (average *R* = 0.91). Download Table S2, XLSX file, 0.1 MB. Copyright © 2018 Xiao et al. 2018 Xiao et al. This content is distributed under the terms of the Creative Commons Attribution 4.0 International license.

### Hemagglutinin gene sequences and SNPs from the H7N9 patient samples.

As shown in Table 5, a number of nonsynonymous SNPs in the hemagglutinin (HA) gene were observed in the patient samples compared to consensus sequences created from all available epizootic H7N9 virus sequences isolated from birds in China in 2013, 2014, and 2015 or compared to all available H7N9 sequences isolated from spillover infections in humans in China in 2013, 2014, and 2015. The 2013 avian H7N9 and human H7N9 consensus HA sequences differed only at codon 235 (aligning to H3 HA codon 226), and this codon forms a part of the HA receptor binding domain (27, 28), with the human consensus sequences from 2013, 2014, and 2015 containing a Q235L mutation. In addition, the avian consensus sequences from 2014 and 2015 also contain a Q235L mutation, which indicates the evolution of this site in avian hosts as well. Among the patient samples, 7 cases (cases 1, 2, 4, 13, 15, 16, and 17) had a Q235L mutation (Table 5; see also Table S1) and 4 cases (cases 5, 6, 19, and 20) had coverage of fewer than 100 reads at this codon, but all of them included Q235L, and the remaining samples had no coverage at HA codon 235. Structural and glycan array analyses have demonstrated that 235L enhances H7 HA binding to human-type α2-6 sialic acids (27, 28), while *in silico* modeling has suggested enhanced binding with Q235I (29). A recent study reported that 3 mutations, H7 HA V195K (equivalent to H3 HA V186K), K202T (H3 K193T), and G237S (H3 G228S), in the receptor binding domain of H7N9 viruses switched specificity from avian-type α2-3 to human-type α2-6 sialic acids (30). In the patient samples, all matched the consensus, V195, K202, and G237.

**FIG 2:**
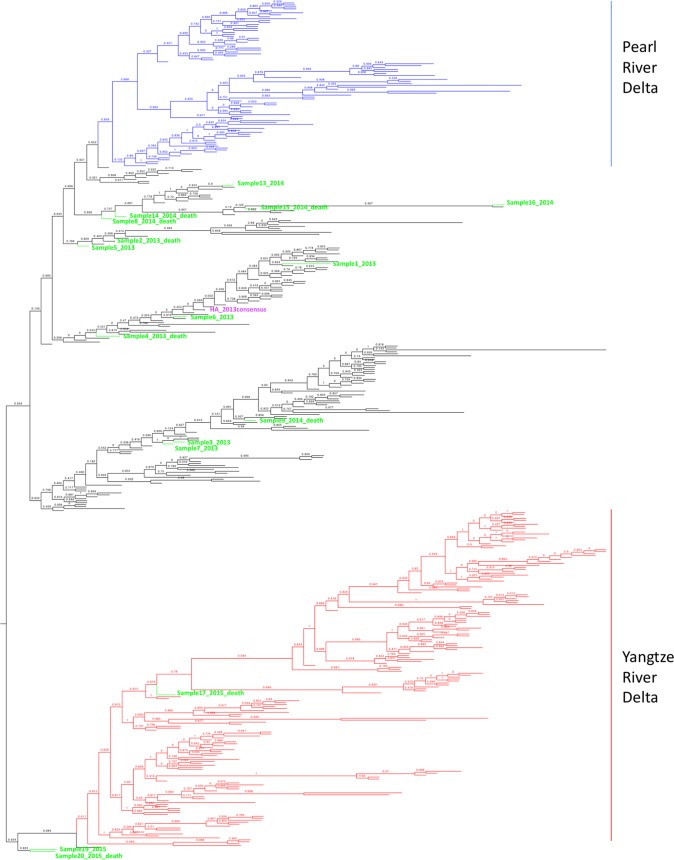
Phylogenetic trees of unique HA segments of human H7N9 in China from 2013 to 2017 and consensus HA sequences (SNP frequency, >50%) of our samples. Approximately maximum-likelihood midpoint-rooted phylogenetic trees with Shimodaira-Hasegawa (SH) values are presented. Purple, reference genome; green, our samples in “sample number_year_outcome” format; blue, viruses in the Pearl River Delta HA lineage; red, viruses in the Yangtze River Delta HA lineage. See Fig. S1 for an expanded form of this tree, in which individual viral taxa are labeled by strain name.

Many additional HA SNPs were also seen in this patient cohort (Table 5). To evaluate the relationship between our samples and other H7N9 sequences in China, we constructed approximately maximum-likelihood phylogenetic trees based on 417 unique human HA sequences from isolates collected in China from 2013 to 2017 together with the consensus sequences (with SNP calls at bases over 50%) from the current study (Fig. 2). Previous studies demonstrated evolutionary diversification of H7N9 HA sequences (7), and with the emergence of the fourth and fifth waves (6, 8), two major lineages have been observed and defined as Yangtze River Delta and Pearl River Delta lineages. Yangtze River Delta lineage isolates have demonstrated reduced cross-reactivity with previously produced candidate vaccine strain H7N9 viruses (6). From the HA trees, it is noted that the sample sequences diversified into different clades, including the Yangtze River Delta lineage (sample 17), despite the fact that the patients were all local residents, treated at only one hospital in Shanghai, with the latest sample from April 2015.

10.1128/mSphereDirect.00462-18.2 FIG S1 Phylogenetic trees of the H7N9 sample sequences from each gene segment. Download FIG S1, PDF file, 0.4 MB. Copyright © 2018 Xiao et al. 2018 Xiao et al. This content is distributed under the terms of the Creative Commons Attribution 4.0 International license.

**FIG 3:**
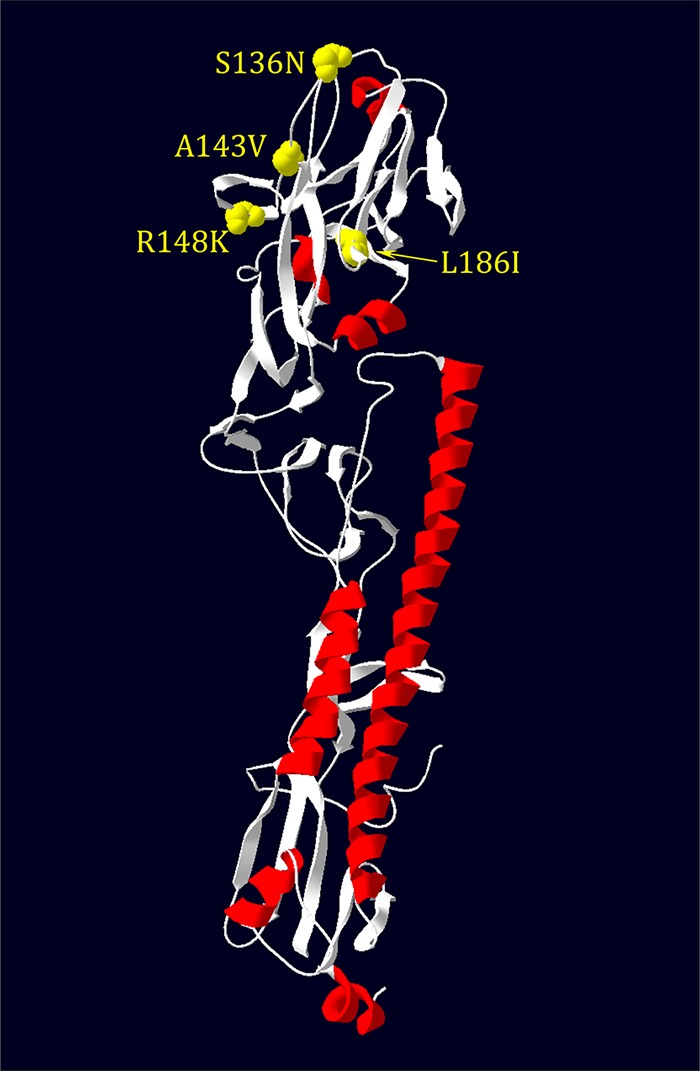
The structure of HA with mutations observed on head regions of sample 17 shown on an H7 HA monomer (A/Anhui/1/2013; see Materials and Methods).

**FIG 4:**
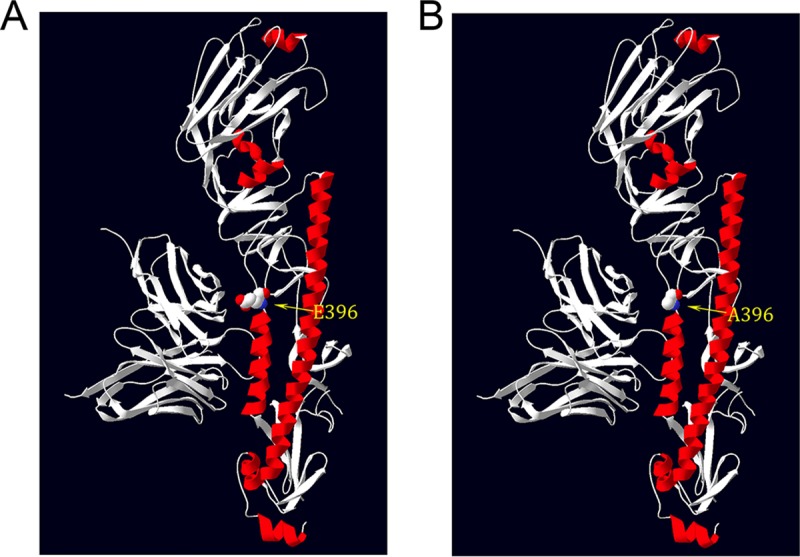
The structure of the HA A396E mutation at the HA stalk related to stalk neutralizing antibody CT149. (A) HA396E. (B) HA396A shown on an H7 HA monomer (A/Anhui/1/2013; see Materials and Methods).

Notable here is the HA sequence from sample 17, representing a wave 3 case from January 2015, which is phylogenetically very similar to the antigenically variant Yangtze River Delta H7N9 sequences isolated in 2016 to 2017 in waves 4 and 5 (8). SNPs identified in sample 17 were notable for the following amino acid changes on the HA head (Table 5): S136N, A143V, R148K, and L186I. These amino acids are highlighted in the head region of the crystal structure of A/Anhui/1/2013 (H7N9) (4R8W) (Fig. 3). While antigenic epitopes on H7 subtype HAs have not been mapped, a computational analysis of H7 HA identified several putative epitopes on the HA head. The first three mutations, S136N, A143V, R148K, are near the T130A (H7 numbering) identified in epitope A (31), while the fourth site, L186I, is between the D183S and I188V changes identified in epitopes C and D, respectively. Computational modeling also identified HA receptor binding mutation Q235L/I as an antigenic epitope (epitope D). Interestingly, these amino acid changes are seen in 2016 to 2017 Yangtze River Delta lineage sequences and are likely to contribute to the antigenic evolution observed in these H7N9 strains compared to earlier human 2013 isolates (6). Changes at H7 HA codons 394 and 396 have been detected in 8 patient samples (samples 8, 13, 14, 15, 16, 17, 19, 20), which were located in the first alpha helix of the HA2 domain. The E396A mutation, in particular, was also observed in more recent Yangtze River Delta lineage isolates, and modeling done here suggests that this change may affect binding of broadly neutralizing stalk antibodies (Fig. 4).

### The remaining viral gene sequences and SNPs from the H7N9 patient samples.

A number of nonsynonymous SNPs were seen in the H7N9 sample neuraminidase (NA) sequences (Table 5), including a R148K change in the catalytic site in sample 1 (equivalent to R152K in N2 subtype NA) (32). At least 15 of the 20 patients were treated with antiviral drugs, primarily oseltamivir (Table 1); however, only one sample (sample 16) showed a change associated with neuraminidase inhibitor (NAI) resistance in these samples (33), which was the most commonly observed resistance mutation, R289K (R292K in N2 subtype NA), with this SNP present at 41% (Table 5). The conserved N9 amino acids associated with the NA sialic acid-binding site (hemabsorption domain) did not differ in the samples studied (34).

SNPs were also observed in the H7N9 polymerase proteins. In PB2, 21 nonsynonymous SNPs were observed in the samples that differed from the reference sequence, including sample 1, which had a K627E change. It has been reported that 627K is associated with human adaptation of avian-origin PB2 (35) and that it was observed in the 1918 pandemic (36) and in some highly pathogenic H5N1 human isolates (37). The human H7N9 PB2 consensus sequence differs from the avian H7N9 consensus sequence at this site as well (Table 5). None of the samples differed at sites 701 and 702, which have been reported to be associated with mammalian adaptation (38). One change was observed in PB1 in sample 9, and 3 changes were observed in samples 6, 7, 8, and 13 in polymerase acidic protein (PA). PA codon 100 is typically a valine in avian IAV sequences, and a V100A change is associated with human adaptation (36). Of the 2013 avian and human H7N9 consensus sequences constructed here, both correspond to V100, and no nonsynonymous SNPs were observed at this codon in all the samples sequenced here. SNPs associated with nonstructural protein PA-X (39) were observed in the samples, but the biological significance of these changes remains unclear. Several other samples had nonsynonymous SNPs in M1, M2, and NS1 (Table 5).

**FIG 5:**
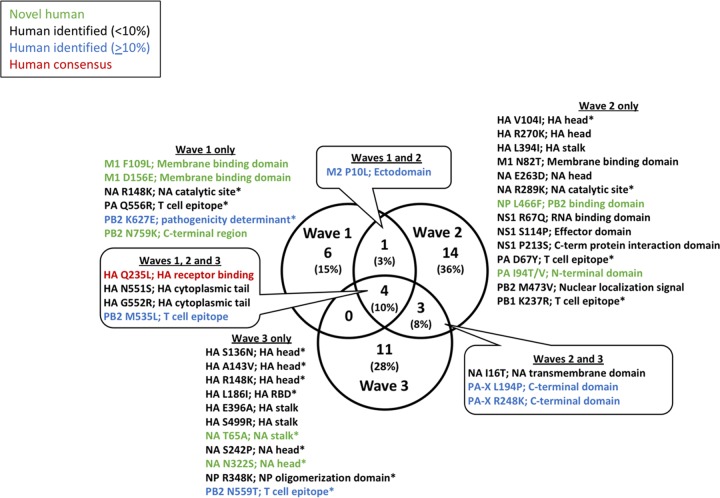
Venn diagram of protein sequences identified in three H7N9 waves between 2013 to 2015 compared to the avian consensus protein sequence. Set analysis was performed on translated nonsynonymous SNPs identified in waves 1, 2, and 3. Novel mutations identified in this study are shown in green; SNPs previously identified in <10% or ≥10% of sequences in the Influenza Research Database are indicated by black text or blue text, respectively. Changes from the avian to the human consensus sequences are shown in red. Percentages of protein sequence changes were rounded to nearest whole-number value. SNPs identified in only a single patient are marked with an asterisk (*). The Venn diagram was created using Venny 2.1 (http://bioinfogp.cnb.csic.es/tools/venny/).

To determine which of the nonsynonymous SNPs could be associated with human adaptation and which were observed during two or more H7N9 epidemic waves, Venn analysis was performed on the translated proteins from nonsynonymous SNPs identified in Table 5 (see also Table S1) compared to the 2013 avian H7N9 protein consensus sequences. As shown in Fig. 5, 15% of H7N9 amino acid changes were identified only in wave 1, 36% were identified only in wave 2, and 28% were unique to wave 3. The populations of amino acid changes identified in only a single wave were dominated by sequences identified at a frequency of <10% in previous human H7N9 cases (black text in the figure), novel human mutations (green text), and one sequence, PB2 K627E (blue text), that had been previously identified in >10% of sequences in the Influenza Research Database as of 19 June 2017. Interestingly, only a single SNP (M2 P10L) was identified in both waves 1 and 2, while 3 SNPs (8% of the total) were present in waves 2 and 3, two of which represented changes in the C-terminal domain of host response regulatory protein PA-X. Finally, 4 SNPs (10% of the total) were identified in all three waves and included changes in HA receptor binding (Q235L), the HA cytoplasmic tail (N551S and G552R), and viral polymerase subunit PB2 (M535L). Of these changes, HA N551S and HA G552R were previously identified in H7N9 human sequences at low (<10%) frequencies, while PB2 M535L was previously identified in ≥10% of human H7N9 sequences. Significantly, the mutation Q235L in the HA receptor binding domain was present in all three waves and represented a change toward the human consensus sequence (denoted with red text in Fig. 5).

### Evidence for reassortment in the H7N9 patient samples.

Phylogenetic analyses were performed by constructing gene segment trees using the approximately maximum-likelihood method (40). Trees were inferred using the compiled H7N9 viral gene segment consensus sequences (proportion of observed SNPs, more than 50%) from the 16 patient samples along with 417 H7N9 viral genomic sequences downloaded from GISAID (http://platform.gisaid.org/epi3/frontend#5277b0). The eight trees are shown in Fig. S1 in the supplemental material. Most trees showed two major clades, often with two subclades in each major clade. Taxa were colored red in the figure for viruses possessing a Yangtze River Delta clade hemagglutinin and blue for viruses possessing a Pearl River Delta clade hemagglutinin. Evidence of gene segment reassortment is apparent among the 8 trees; for example, several viruses with Pearl River Delta clade HA genes (colored in blue) are contained in the Yangtze River Delta clade (colored in red) of the neuraminidase tree. Following the taxa within the trees, it is possible to observe frequent reassortment within each gene segment, as previously reported (41).

## DISCUSSION

Human infections with avian IAV H7 subtype viruses are rare but have been reported before and are usually associated with exposure to poultry (42–44). Most of the previously reported cases of H7 IAV infection in humans have caused only mild or moderate illness (43–45), except for one fatal case resulting from a highly pathogenic avian H7N7 IAV infection in the Netherlands (46). In contrast, the current avian IAV H7N9 epizootic outbreak in China has been much more severe, with more than 90% of the confirmed H7N9 patients being hospitalized with pneumonia or respiratory failure and with a CFR of 39%. This suggests that the 2013 lineage avian H7N9 virus may be more pathogenic in humans than the H7 strains responsible for past IAV epizootic outbreaks (47). Human H7N9 virus infections have also exhibited age-specific and sex-specific morbidity and mortality patterns. The median age of the confirmed H7N9 patients was 58 years. A very few H7N9 cases have occurred in children, teenagers, and young adults. Further, most of the severe and fatal cases occurred in middle-aged and older men rather than in women (48). However, females in their reproductive years have displayed an increased tendency to die of H7N9 influenza in comparison to males (49). In this patient cohort, the observed CFR was high, with an overall fatality rate of at least 55% (some patient outcomes were not available); however, no correlation could be made between outcome and the SNPs identified.

In this study, all 20 patient samples were sequenced and mapped to the H7N9 reference genome. On the basis of the negative-control results with respect to reads mapped to H7N9 genome, 4 samples were eliminated from further analysis. Among the remaining 16 samples, the total numbers of mapped reads ranged from 105,000 to over 178 million and the range of H7N9 genome coverage was 57.7% to 99.5% (Table 3). The differences in the mapped read numbers and coverage percentages depended on the viral RNA concentration and the quality of viral RNA in the different samples. Viral RNA degradation during sample preservation and/or library preparation would also likely decrease coverage.

Previous studies have demonstrated that human host factors are likely important in determining outcome during influenza virus infections. For example, more than 200 host factors are required for influenza virus replication as demonstrated by genome-wide RNA interference screening (50, 51). Other studies showed that interferon-inducible transmembrane 3 (IFITM3) may be critical for defending the host against IAV *in vivo* (52, 53). Even administration of influenza vaccinations at different times of day has been shown to affect the development of host antibody responses (54). A recent study of whole-genome exon capture and sequencing of 18 H7N9 patients identified 21 genes that were highly associated with H7N9 influenza virus infection, some of which may be associated with H7N9 influenza susceptibility (55). Thus, different hosts, different health conditions, and even different times of infection could affect the evolution of IAV.

In addition, when the SNPs identified in the current study were compared to the precomputed SNPs called from human H7N9 sequences in the Influenza Research Database (25), we found that 39.2% (178/454) of the SNPs from the samples had not been previously reported. One reason for this could be that deep-sequencing technology was applied to direct sequencing of virus from clinical samples here, which allowed investigation of infected viral populations, including identification of quasispecies. These methods are now becoming more common. Historically, the majority of viral sequences in IRD or GenBank were likely initially cultured and then subjected to PCR amplification and Sanger sequencing to generate consensus genome sequences, some of which may have also acquired culture adaptations (56).

As seen in other human H7N9 isolates, the HA sequences determined in this study showed the change in the receptor binding domain, Q235L/I (H3 Q226L/I) (28, 29, 57, 58), that has been reported to enhance binding to human-type α2-6 sialic acids (59). Among all the 16 samples examined here, 11 samples with read coverages possessed Q235L, whereas, among the 417 unique HA sequences downloaded from GISAID, only 9 strains possessed Q235I. It will be interesting to see if this mutation is more commonly observed in future human H7N9 isolates. Of the 3 mutations recently modeled *in vitro* to switch HA receptor specificity to α2-6 sialic acids (30), none were observed in this study.

Sample 17 possessed several nonsynonymous SNPs on the HA head domain that are shared with antigenically variant Yangtze River Delta lineage viruses isolated in waves 4 and 5 in 2016 to 2017 (Fig. 3) and likely reflect some changes related to the antigenic evolution in these H7N9 viruses compared to earlier (2013) isolates (6). Thus, compared to conventional sequencing methods, deep-sequencing strategies may aid in the identification of variant viruses with potentially significant changes associated with human adaptation or with antigenicity acquired more readily and earlier. Since there is no evidence of efficient human-to-human transmission or significant population immunity against H7N9 in humans, antigenic drift, as observed in sample 17 and related Yangtze River Delta lineage viruses, is likely occurring in the avian reservoir and may reflect widespread use of IAV vaccines in poultry in China (60).

Another nonsynonymous HA mutation identified in the samples is E396A in the HA stalk region. This mutation was observed at a rate of over 98% in samples 17, 19, and 20. Structural modeling based on the cocrystal structure of the H7 HA with the stalk neutralizing antibody CT149 (61) supports the idea that the E396A mutation could affect HA stalk antibody binding in this region (Fig. 4). Among the 417 unique HA sequences downloaded from GISAID, 171 possessed an A396; 60 of those strains were from the year 2017, 43 from 2016, 50 from 2015, and 18 from 2014. This might indicate an antigenic drift trend, but additional experiments are needed to evaluate whether this change would affect broadly neutralizing stalk antibodies.

In this study, 15 of our 20 patients were treated with oseltamivir (Table 1). It has been reported that neuraminidase mutations E119V, I222V, D197N/E, and R292K (N2 numbering) have been commonly detected in H3N2 and type B viruses (62–66) after neuraminidase inhibitor (NAI) treatment. Moreover, corresponding mutations E119V, I222K, and R292K in H7N9 reduced the inhibitory activity of oseltamivir in ferrets (67). In our samples, only R289K (N2 R292K) was detected in patient 16 (59% R and 41% K) (Table 5), but the antiviral treatment status of this patient is unknown. The samples from 3 patients (patients 14, 19, and 20) did not have adequate read coverage at this position, but the remaining samples were all R289 despite most of the patients (except patients 10, 11, 13, and 14, who had incomplete medical records) having received oseltamivir treatment (Table 1). Even lowering the SNP call stringency to a 5% difference compared to the reference, no additional oseltamivir resistance mutations were found in the patient samples. The explanation could be the sampling took place in each case on or right after the dates of the antiviral treatments in all our patients, except patient 12, whose sampling date was 8 days after the antiviral treatment date.

It has been shown that some amino acid changes in the viral polymerase complex of IAV are very important for mammalian adaptation. For example, PB2 E627K and D701N changes are critical for mammalian adaptation (35–37, 68, 69) and the changes could increase polymerase activity and viral replication in mammalian cells and pathogenicity of H7N9 viruses in mice (70–72). It was also found that E627K and D701N mutations in H7N9 could readily occur during transmission of the virus among ferrets via direct physical contact (73). Among our samples, only sample 1 displayed a mixture of E627K mutations (68% E, 32% K). For the PB2 701 site, the samples from all patients with adequate coverage at this site possessed D701.

Interestingly, among the samples, 4 of the key SNPs were present in patients from all 3 waves (Fig. 5), including changes in the viral polymerase subunit PB2 (M535L). Other than being mapped to T cell epitopes, it is not known whether these observed PB2 changes or the change in NP would enhance replication or transmissibility in mammals. Further studies are needed to understand the significance of these changes.

Critical for prepandemic surveillance efforts is timely recognition of the emergence of mutations that indicate the appearance of a viral genotype with an increased capacity for forward transmission. In addition to the polymerase mutations that facilitate mammalian viral replication, such as PB2 E627K (35, 74), as well as the changes in the HA receptor binding domain that increase the affinity for α2-6 sialic acids of the human upper respiratory tract (75, 76), our analysis showed that the PB2 M535L change was identified in multiple patients and was present during all three H7N9 waves between 2013 and 2015. Additionally, changes in the HA cytoplasmic tail (N551S and G552R) were also identified in these three successive waves. The cytoplasmic tail of HA is a site for cysteine acylation (77, 78) and plays an important role during infection by modulating membrane fusion activity during viral assembly and budding (79–81). While it is unclear what role N551S and G552R play in infection, it is tempting to speculate that these changes could be associated with increased viral budding in humans either through acylation-dependent or -independent processes.

To our knowledge, this is the first report of deep-sequencing analysis of H7N9 IAV directly from clinical samples of patients from three consecutive years without initial viral culture or influenza virus-specific RT-PCR analyses. Applying whole-transcriptome amplification methods, specific enrichment for H7N9 influenza reads, and deep-sequencing methods, we were able to both investigate the consensus sequences of the viral genome and identify low-abundance SNPs. A possible advantage of the methods described in this report, including enrichment of viral RNA by specific probe hybridization, is the ability to obtain sequences from samples with low amounts of or degraded viral RNA. Although the samples analyzed in the present study were all from patients treated at a single hospital in Shanghai, SNP analyses showed that the viruses contained signatures of different evolving H7N9 clusters (6–8), including a virus isolate from a patient infected in 2015 with likely antigenic changes to the HA that are shared with more recently isolated Yangtze River Delta lineage isolates. In addition, we identified 178 SNPs with 106 nonsynonymous changes in H7N9 that have not been reported in the Influenza Research Database. However, further investigation is still needed to determine the function of these newly identified SNPs with respect to virus replication, host adaptation, transmission, or pathogenesis.

## MATERIALS AND METHODS

### Patient information and sample collection.

The samples from 20 patients infected by avian influenza virus H7N9 studied here were collected in Shanghai, China, between 2013 and 2015. All 20 patients were diagnosed with severe community-acquired pneumonia (CAP). Throat swabs were collected from each patient during the inpatient stay and stored in viral transport medium (VTM) (Yocon, China). RNA was extracted from the swab samples using a nucleic acid extraction kit (QIAamp viral RNA minikit; Qiagen, USA). The presence of influenza A/H7N9 virus RNA was tested using the RT-PCR method recommended by the World Health Organization (82). The samples were collected and processed for viral sequence analysis following elimination of personally identifiable information. The sequence analyses of H7N9 virus genomes performed at the National Institutes of Health (NIH) were determined to represent an excluded study, i.e., one not requiring Institutional Review Board (IRB) approval by the NIH Office of Human Subjects Protections (exemption number 13268).

### Target enrichment probe design.

Enrichment probes were designed by Agilent Technologies (Santa Clara, CA) using A/Hangzhou/1/2013 (H7N9) as the reference sequence. The biotin-labeled RNA probes were 120 bases in length with 5-base spacing density, resulting in a total of 2,034 overlapping probes spanning the H7N9 genome.

### Library construction and sequencing.

For negative-control experiments, HeLa cell total RNA was obtained from TaKaRa (TaKaRa Bio USA, Inc., Mountain View, CA). For positive-control experiments, the hemagglutinin (HA) gene segment (KC853766.1) from A/Hangzhou/1/2013 (H7N9) was synthesized and cloned into the pBluescript II KS(-) SacI site by GenScript (Piscataway, NJ). Plasmid was linearized by NotI (NEB, Ipswich, MA) digestion, and RNA was transcribed using a HiScribe T7 high-yield RNA synthesis kit (NEB, Ipswich, MA) following the manufacturer’s instructions. RNA from the HeLa cells, from the T7 promoter-transcribed HA plasmid, and from the isolated total RNA from patient sample throat swabs was amplified using Ovation RNA-Seq system V2 from NuGEN (NuGEN, San Carlos, CA) following the kit specifications. Each sample, containing 5 μl of total RNA, was used as input for Ovation RNA-Seq system V2. The amplified total cDNAs were analyzed using an Agilent 2100 Bioanalyzer with an Agilent high-sensitivity DNA kit (Agilent Technologies, Santa Clara, CA) and sheared to 150 bp using a Covaris S2 instrument (Covaris, Woburn, MA). Following this, approximately 300 ng amplified cDNA was used to make each Illumina sequencing library using an Agilent SureSelect^XT^ target enrichment kit (Agilent, Santa Clara, CA) for Illumina multiplex sequencing and the protocol for 200-ng DNA samples. Enriched Illumina sequencing libraries were analyzed on an Agilent 2100 Bioanalyzer using an Agilent high-sensitivity DNA kit. Libraries were then clustered on an Illumina cBot instrument and sequenced on Illumina GAIIx or HiSeq machines according to the instructions of the manufacturer (Illumina, San Diego, CA). In this study, more than 2.8 billion reads and a total of more than 323 Gb of sequences were generated.

### Data analysis.

A consensus sequence derived from all available H7N9 human strains obtained in China in 2013 was used as the reference genome and was generated at the Influenza Virus Resource site (https://www.ncbi.nlm.nih.gov/genomes/FLU/Database/nph-select.cgi?go=database) (83) on 16 November 2017. This reference sequence is called the 2013 human H7N9 consensus in this study (the sequences are listed in Text S1 in the supplemental material). Amino acid consensus sequences from avian and human H7N9 isolates from 2013, 2014, and 2015 were generated similarly (the sequences are listed in Text S1). Reads were mapped to Bowtie2 (version 2.2.5; http://bowtie-bio.sourceforge.net/bowtie2/index.shtml) indexed to the consensus sequence of all H7N9 human strains in China in the 2013 genome using Tophat2 (release 2.0.13; http://ccb.jhu.edu/software/tophat/index.shtml) downloaded from the Center for Computational Biology, Johns Hopkins University (http://ccb.jhu.edu/) (84). SAMtools mpileup (version 2.1.0) was used to make SNP calls, and the minimum base Phred quality score was 25 (85). The reported SNP calls needed to satisfy the indicated criteria at the SNP position, similarly to those described in a previous study (86), were as follows: (i) more than 100 reads at that position; (ii) reads with SNP calls from both read directions; (iii) a SNP call not at the first or last base of all the SNP-containing reads; and (iv) different bases representing more than 10% of the aligned reads.

10.1128/mSphereDirect.00462-18.1 TEXT S1 H7N9 consensus sequences generated and used for this study. Download Text S1, DOCX file, 0.03 MB. Copyright © 2018 Xiao et al. 2018 Xiao et al. This content is distributed under the terms of the Creative Commons Attribution 4.0 International license.

Multiple-sequence alignment was performed using MUSCLE (87) version 3.8.31, and midpoint-rooted phylogenetic trees using the approximately maximum-likelihood method were constructed with FastTree version 2.1.8 with the Jukes-Cantor + CAT model (40) and displayed in FigTree version 1.4.3 (http://tree.bio.ed.ac.uk/software/figtree/) and included Shimodaira-Hasegawa test values. Other human H7N9 sequences from the outbreak samples collected in China in 2013 to 2017 and used in this study were downloaded from the Global Initiative on Sharing All Influenza Data (GISAID; http://platform.gisaid.org/epi3/frontend#355fbd) database on 6 April 2017. Identical sequences from the HA segments were eliminated before alignment. The strain names used in the HA tree were used to identify the sequences to make phylogenetic trees for other IAV H7N9 gene segments. In the sample sequences, at nucleotide positions with read coverage but with fewer than 100 reads, bases from the precomputed human H7N9 consensus sequence from each segment were used to assemble contigs.

### Protein structure.

The crystal structure of the H7 hemagglutinin from A/Anhui/1/2013 in complex with neutralizing antibody CT149 (4R8W) (61) was downloaded from the Research Collaboratory for Structural Bioinformatics (RCSB) Protein Data Bank (PDB) (http://www.rcsb.org/pdb/home/home.do) (88), and HA amino acid changes were made and viewed with Swiss-PdbViewer (89) or with the protein structure viewer in the Influenza Research Database (90).

### Accession number(s).

All sequences have been deposited as a series with accession no. SRR7444529 to SRR7444548 at the GenBank SRA database.
