# Supplementary figures and images for: Deep Sequencing of H7N9 Influenza A Viruses from 16 Infected Patients from 2013 to 2015 in Shanghai Reveals Genetic Diversity and Antigenic Drift
Source: mSphere. 2018 Sep 19;3(5):e00462-18. doi: 10.1128/mSphereDirect.00462-18 (PMC6147129; doi:10.1128/mSphereDirect.00462-18)

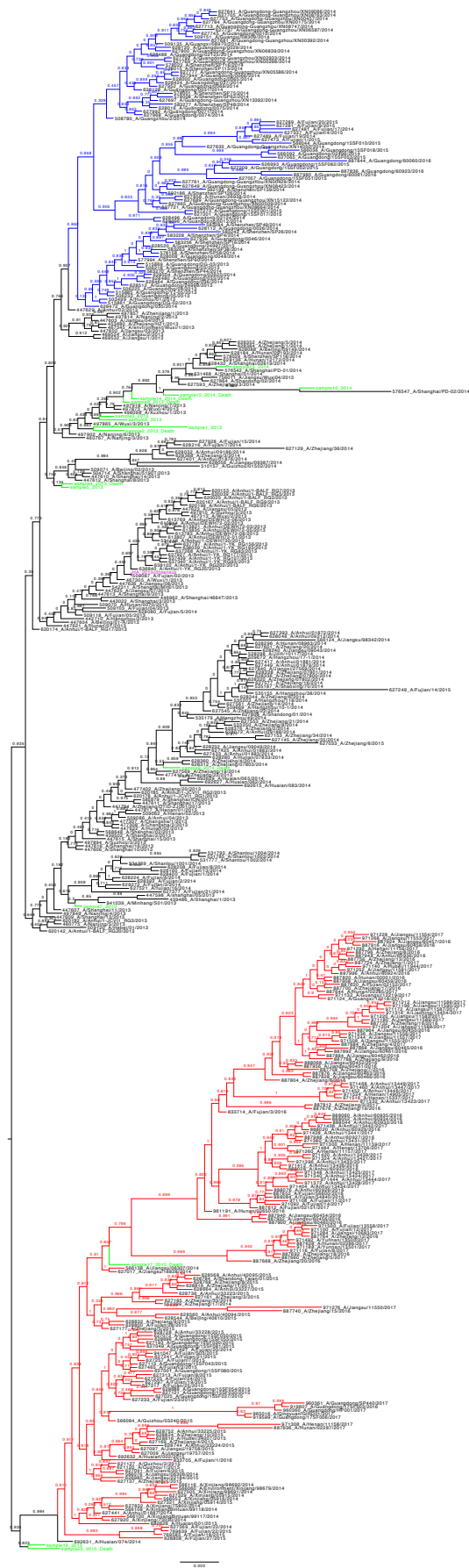

HA

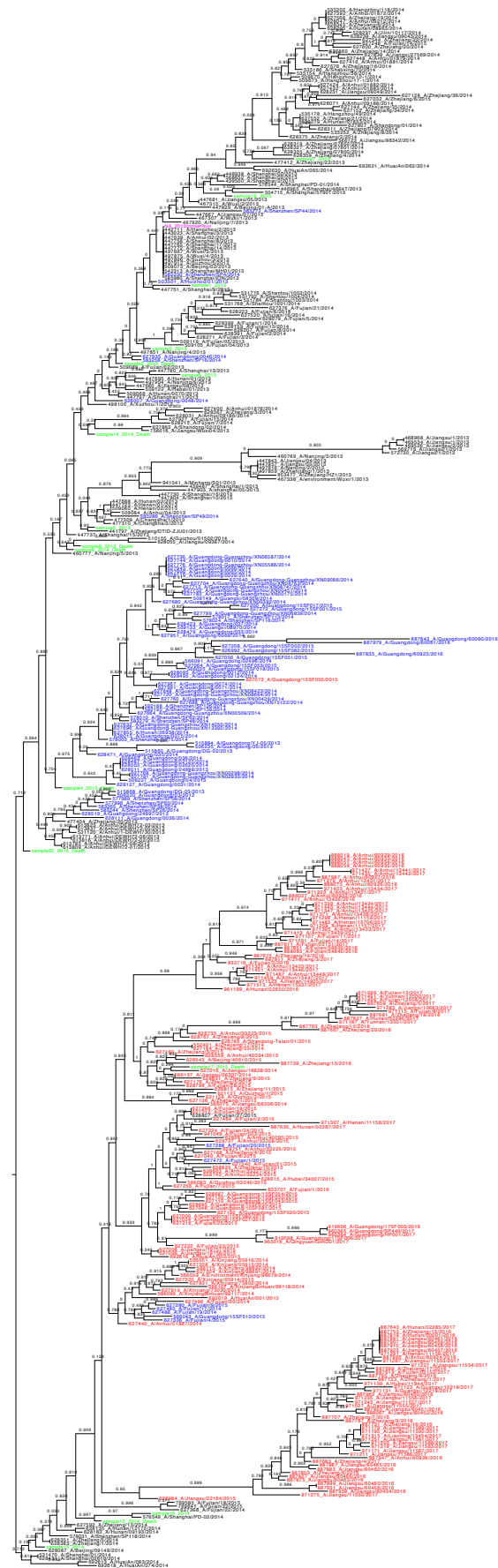

NA

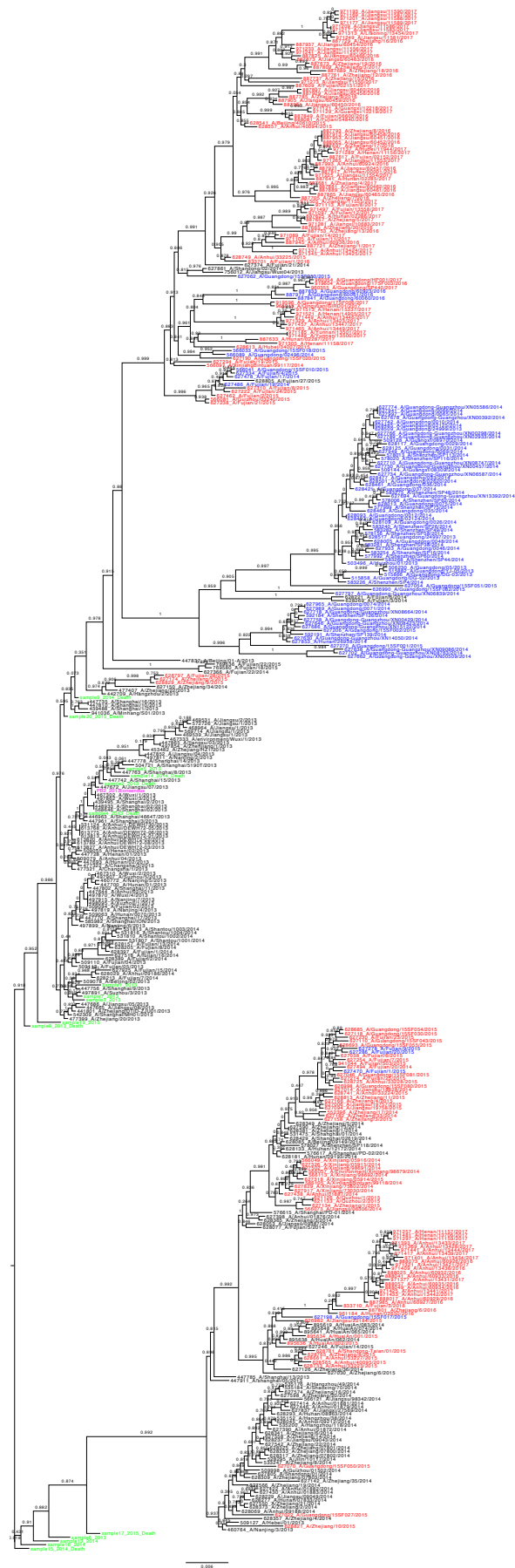

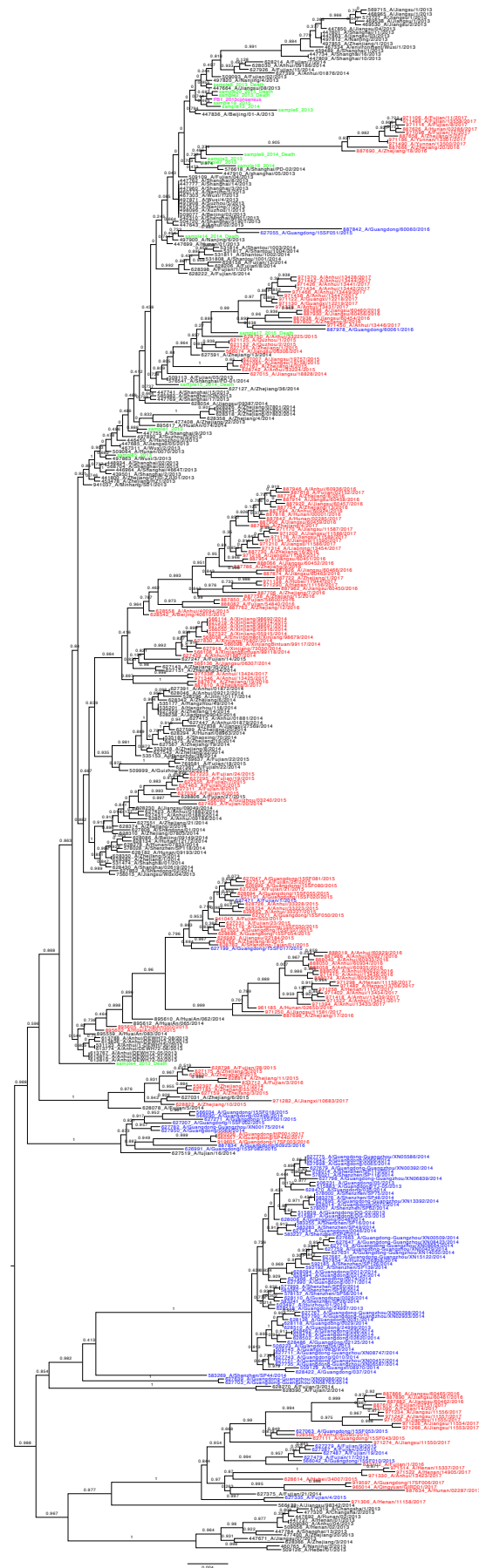

PB1

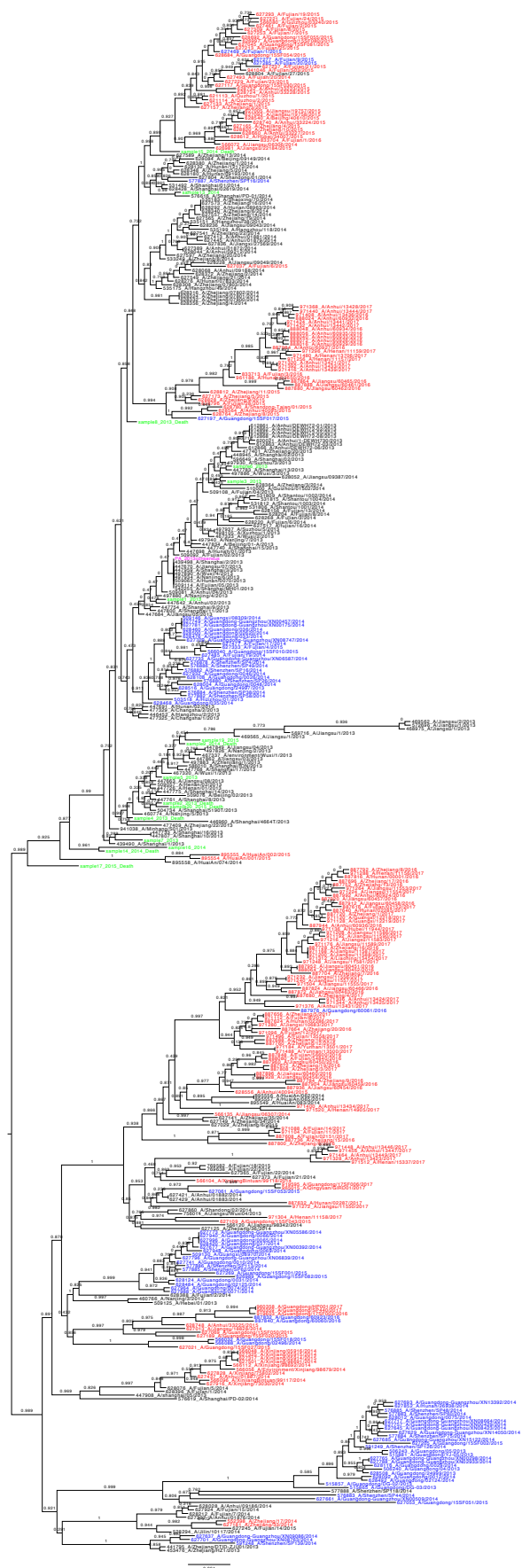

PA

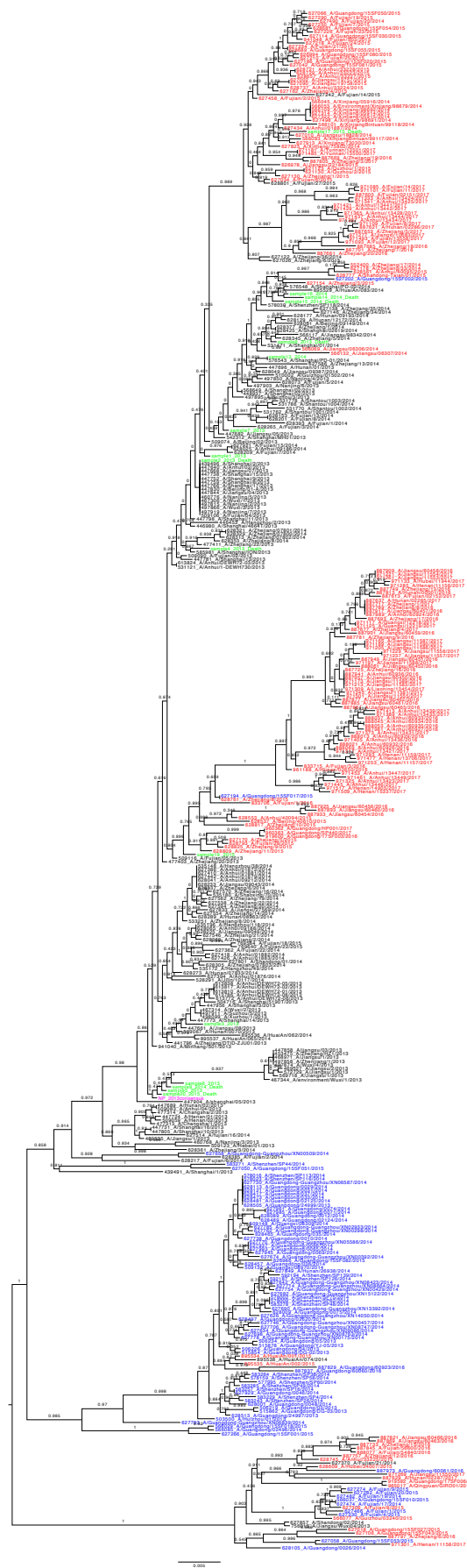

NP

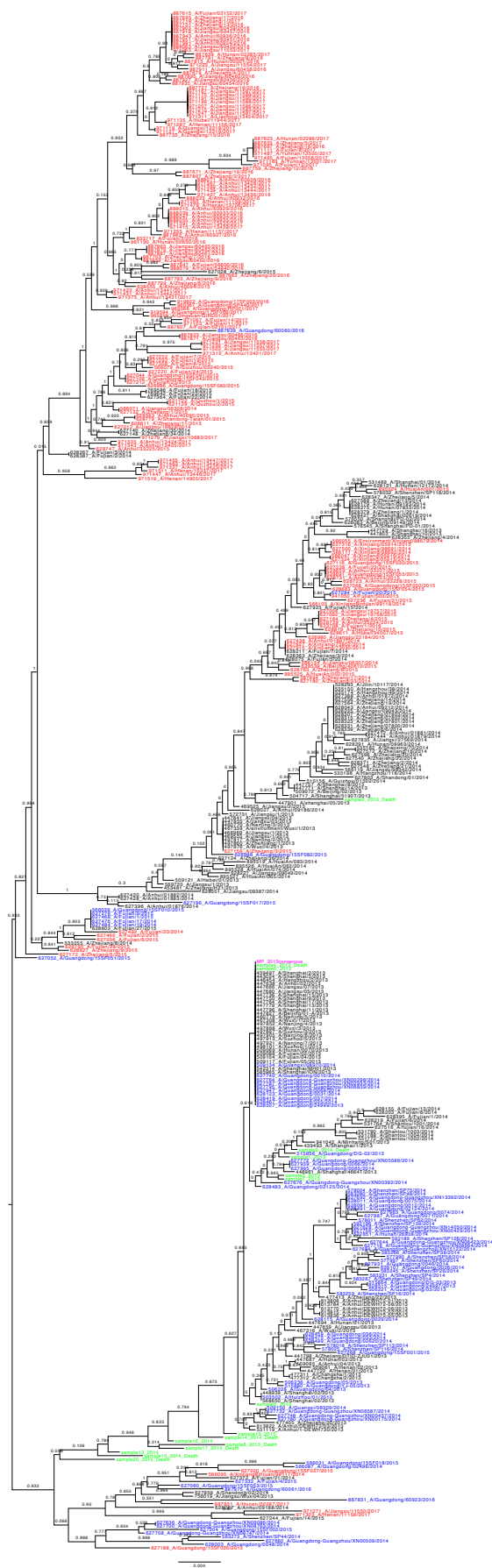

MP

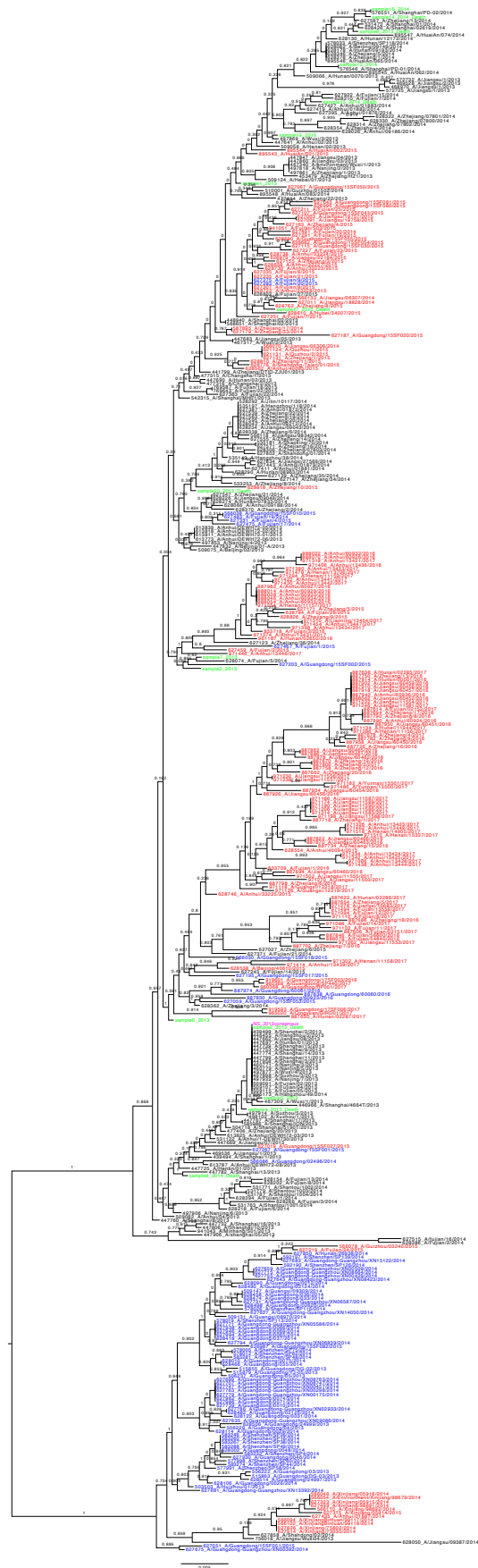

NS

Supplement: FIG S1 [file sph005182648sf1.pdf]
