# Supplementary material for: Deep Sequencing of H7N9 Influenza A Viruses from 16 Infected Patients from 2013 to 2015 in Shanghai Reveals Genetic Diversity and Antigenic Drift
Source: mSphere. 2018 Sep 19;3(5):e00462-18. doi: 10.1128/mSphereDirect.00462-18 (PMC6147129; doi:10.1128/mSphereDirect.00462-18)
Supplement: TEXT S1 [file sph005182648s1.docx]

**Supplemental File**

**2013 H7N9 Human Consensus Nucleotide Sequences in China (Used for sequence alignment):**

>PB2_2013consensus

ATGGAAAGAATAAAAGAACTAAGAGATTTGATGTCACAGTCTCGCACTCGCGAGATACTG

ACAAAAACAACTGTGGACCATATGGCCATAATCAAGAAATATACATCAGGAAGACAGGAG

AAGAATCCTGCCCTTAGGATGAAGTGGATGATGGCAATGAAATATCCAATTACGGCAGAC

AAAAGGATAATGGAGATGATCCCGGAAAGAAATGAGCAAGGTCAGACCCTTTGGAGCAAG

ACAAATGATGCTGGATCAGACAGAGTGATGGTGTCACCTCTGGCTGTGACGTGGTGGAAC

AGAAATGGACCAACGACAAGCACAGTCCATTATCCAAAGGTCTATAAAACCTATTTTGAA

AAGGTCGAAAGGCTAAAACATGGAACCTTCGGCCCCGTTCACTTCAGAAACCAGGTTAAA

ATACGCCGCAGGGTCGACATAAACCCGGGCCATGCAGATCTTAGTGCTAAAGAAGCACAG

GATGTCATCATGGAGGTCGTATTCCCAAACGAAGTTGGAGCCAGAATATTGACATCAGAG

TCACAGTTAACGATTACCAAGGAAAAGAAGAAGGAGCTTCAGGACTGCAAAATTGCCCCT

TTAATGGTGGCTTACATGTTGGAGAGAGAACTGGTTCGCAAAACAAGATTCCTACCAGTA

GCTGGAGGGACAAGCAGCGTGTATATCGAGGTGTTGCATTTGACCCAAGGGACCTGCTGG

GAGCAAATGTACACACCGGGAGGGGAAGTGAGAAATGATGATGTTGATCAGAGTTTAATT

ATTGCTGCTAGAAATATTGTTAGAAGAGCAACAGTATCAGCAGACCCGTTGGCTTCGCTT

TTGGAGATGTGCCATAGTACACAGATTGGCGGGGTTAGGATGGTTGACATCCTTAGACAA

AACCCAACAGAAGAACAGGCTGTGGATATATGTAAGGCAGCAATGGGTCTAAGGATCAGT

TCATCCTTCAGCTTTGGAGGTTTCACTTTCAAAAGGACAAGTGGGTCATCTGTCAAAAGG

GAAGAAGAAGTGCTCACAGGCAACCTCCAAACATTGAAAATAAGAGTACATGAAGGATAT

GAGGAATTCACAATGGTCGGGCGAAGAGCAACAGCCATTCTAAGGAAAGCAACCAGAAGA

CTGATCCAACTGATAGTGAGTGGGAAAGACGAGCAATCAATCGCCGAGGCAATCATAGTG

GCAATGGTATTCTCACAAGAGGATTGTATGATAAAGGCAGTGAGAGGTGATTTGAACTTT

GTCAACAGAGCAAACCAGCGGCTAAATCCCATGCATCAACTCCTGAGGCATTTCCAAAAG

GATGCAAAGGTCCTGTTTCAAAACTGGGGAATTGAACCCATTGACAATGTAATGGGGATG

ATCGGAATATTGCCTGACATGACCCCCAGCACAGAGATGTCATTGAGAGGAGTGAGAGTT

AGTAAAATGGGAGTAGATGAATATTCCAGCACTGAGAGAGTGGTCGTGAGTATTGATCGT

TTCTTGAGGGTCCGAGACCAGAGGGGAAACGTACTCCTGTCTCCTGAAGAGGTTAGTGAA

ACACAGGGAACAGAAAAGCTGACTATAACATATTCATCGTCCATGATGTGGGAGATCAAT

GGTCCGGAATCAGTGCTAGTTAACACATATCAATGGATCATTAGAAATTGGGAAAATGTA

AAGATTCAATGGTCCCAAGATCCTACAATGCTATACAATAAGATGGAATTTGAACCCTTT

CAATCCCTAGTGCCTAAAGCTGCCAGAGGCCAATATAGTGGGTTCGTGAGGGTTCTATTC

CAACAGATGCGTGACGTACTGGGAACATTTGACACTGTCCAAATAATAAAGCTATTACCA

TTTGCAGCAGCCCCGCCGAAGCAGAGTAGGATGCAGTTCTCTTCTCTAACTGTGAATGTG

AGGGGTTCCGGAATGAGAATAGTTGTGAGAGGCAATTCTCCTGTGTTCAACTACAACAAG

GCAACCAAGAGGCTTACGGTGCTTGGAAAGGATGCAGGTGCATTGATGGAAGACCCCGAT

GAGGGAACAGCAGGAGTGGAATCTGCGGTATTGAGGGGATTTCTGATTCTGGGCAAAGAA

GACAAAAGATATGGGCCAGCATTGAGCATCAACGAATTGAGCAATCTTGCGAAAGGAGAG

AAGGCTAATGTGTTGATAGGGCAAGGAGACGTTGTGTTGGTGATGAAACGGAAACGGGAC

TCTAGCATACTTACTGACAGTCAGACAGCGACCAAAAGGATTCGGATGGCCATCAATTAA

>PB1_2013consensus

ATGGATGTCAATCCGACTTTACTTTTCTTGAAAGTGCCAGTGCAAAATGCTATAAGTACC

ACTTTCCCTTATACTGGAGACCCTCCATACAGCCATGGAACAGGAACAGGATACACCATG

GACACAGTCAACAGAACACATAAATACTCAGAAAAAGGAAAGTGGACAACGAACACAGAG

ACTGGAGCACCCCAACTCAATCCAATTGATGGACCATTACCTGAGGACAACGAGCCGAGT

GGGTATGCACAAACGGATTGTGTATTGGAAGCAATGGCTTTCCTTGAAGAATCTCACCCA

GGGATCTTTGAAAACTCGTGTCTCGAAACGATGGAAATTGTTCAGCAAACAAGAGTGGAT

AAACTGACCCAAGGCCGCCAGACCTATGACTGGACGTTGAATAGAAATCAGCCGGCTGCT

ACCGCATTGGCCAACACTATAGAGGTATTCAGATCGAATGGCCTGACAGCCAATGAATCA

GGAAGGTTGATCGATTTCCTCAAGGACGTGATGGATTCAATGGATAAGGAAGAAATGGAG

ATTACAACACATTTCCAGAGGAAGAGGAGAGTGAGGGACAACATGACCAAGAAAATGGTC

ACACAGAGAACAATAGGAAAGAAAAAACAAAGACTGAACAAAAGGAGCTACCTAATAAGA

GCACTGACATTGAACACAATGACAAAGGATGCTGAAAGAGGCAAGCTGAAAAGGAGGGCA

ATCGCAACACCCGGGATGCAAATCAGAGGATTCGTGTATTTTGTAGAAGCACTAGCGAGG

AGCATCTGTGAGAAACTTGAGCAATCTGGCCTCCCTGTCGGAGGGAATGAGAAGAAAGCT

AAATTGGCAAATGTTGTGAGGAAGATGATGACTAATTCACAAGATACAGAGCTCTCCTTC

ACAATTACTGGGGACAACACCAAATGGAATGAGAATCAAAACCCCCGGATGTTTCTAGCA

ATGATAACATACATCACAAGAAACCAGCCAGAATGGTTTAGAAATGTCTTAAGCATTGCT

CCTATAATGTTCTCAAACAAGATGGCGAGATTAGGAAAAGGGTACATGTTCGAAAGTAAG

AGTATGAAGTTACGGACACAAGTACCAGCGGAAATGCTCGCAAATATTGACCTGAAATAC

TTCAACAAATCAACAAGAGAGAAAATCGAGAAAATAAGACCTCTACTGATAGATGGCACA

GCCTCATTGAGTCCTGGAATGATGATGGGCATGTTCAACATGTTGAGTACAGTCTTAGGA

GTTTCAATTCTGAATCTCGGGCAGAAGAAGTACACCAAAACCACATATTGGTGGGACGGA

CTCCAATCCTCAGATGACTTCGCCCTCATAGTGAATGCACCGAATCATGAGGGAATACAG

GCAGGAGTAGATAGGTTCTATAGAACCTGCAAATTAGTTGGGATAAACATGAGCAAGAAG

AAATCCTACATAAATCGGACAGGAACATTCGAATTCACAAGCTTTTTCTACCGCTATGGA

TTCGTAGCTAACTTCAGTATGGAGTTGCCCAGTTTTGGAGTGTCCGGGATTAATGAGTCA

GCTGACATGAGCGTTGGTGTTACAGTAATAAAGAACAATATGATAAACAACGATCTTGGA

CCAGCAACAGCCCAAATGGCCCTTCAGCTATTTATCAAAGACTACAGATACACATACCGA

TGTCACAGGGGTGATACGCAAATTCAAACGAGGAGAGCATTCGAGCTGAAGAAGCTGTGG

GAGCAGACCCGTTCGAAGGCAGGACTGTTGGTTTCAGATGGAGGGCCAAACCTGTACAAT

ATCCGGAACCTCCACATTCCAGAGGTCTGCTTGAAATGGGAATTGATGGATGAAGACTAC

CAAGGCAGGTTGTGTAATCCTATGAACCCGTTTGTCAGTCATAAGGAAATTGATTCAGTC

AACAATGCTGTGGTGATGCCAGCTCATGGCCCAGCCAAAAGCATGGAGTATGATGCCGTT

GCAACCACACATTCATGGATTCCTAAGAGGAATCGCTCCATTCTCAACACCAGCCAAAGG

GGGATTCTTGAGGACGAACAGATGTACCAGAAGTGCTGCAACCTATTCGAAAAGTTCTTC

CCCAGCAGTTCGTACAGGAGGCCAGTTGGAATTTCCAGCATGGTGGAGGCCATGGTGTCT

AGGGCCCGAATTGATGCACGAATTGACTTCGAATCTGGAAGGATTAAGAAAGAAGAGTTT

GCTGAGATCATGAAGATCTGTTCCACCATTGAAGAGCTCAGACGGCAAAAATAA

>PA_2013consensus

ATGGAAGACTTTGTGCGACAGTGCTTCAATCCAATGATCGTCGAGCTTGCGGAAAAGGCA

ATGAAAGAATATGGGGAAGATCCGAAAATCGAAACAAACAAATTCGCATCAATATGCACA

CACTTAGAAGTCTGCTTCATGTACTCTGATTTCCACTTCATCGACGAACGAGGCGAATCA

ACTATAATAGAATCTGGCGATCCAAATGCGCTGCTGAAACACCGATTTGAAATAATCGAA

GGGAGAGACCGAACAATGGCCTGGACAGTGGTGAATAGTATCTGCAACACCACAGGAGCC

GAAAAACCCAAATTTCTCCCGGATCTGTATGACTACAAGGAAAACCGTTTCATTGAAATT

GGAGTGACGAGGAGGGAAGTCCACATATATTACCTAGAGAAAGCCAATAAAATAAAATCC

GAGAAGACACACATCCATATTTTTTCATTCACTGGAGAAGAGATGGCCACCAAAGCAGAT

TACACTCTTGACGAAGAAAGCAGGGCAAGAATCAAAACCAGGCTGTTCACCATAAGGCAG

GAAATGGCCAGCAGGGGTCTATGGGATTCCTTTCGTCAGTCTGAAAGAGGCGAAGAAACA

ATTGAAGAAAGATTTGAAATCACAGGAACCATGCGCAGGCTTGCCGACCAAAGTCTCCCA

CCGAACTTCTCCAGCCTTGAAAACTTTAGAGCCTATGTGGATGGATTCGAACCGAACGGC

TGCATTGAGGGCAAGCTTTCTCAGATGTCAAAAGAAGTGAACGCCAGAATTGAGCCATTT

CTAAGAACAACACCACGCCCTCTCAGATTGCCTAATGGGCCTCCCTGCTCTCAGCGGTCG

AAATTCTTGCTGATGGATGCTCTGAAATTAAGCATTGAGGACCCGAGCCACGAAGGGGAG

GGGATACCGCTATATGATGCGATCAAATGCATGAAAACGTTCTTCGGGTGGAAAGAGCCC

AACATTATCAAACCACATGAGAAAGGCATAAACCCCAATTATCTCCTGACTTGGAAGCAG

GTGCTAGCAGAACTTCAGGACATTGAAAATGAAGAGAAGATTCCAAGGACAAAGAACATG

AAGAAAACAAGCCAATTAAAGTGGGCACTCGGTGAGAACATGGCACCGGAGAAGGTGGAC

TTTGAGGATTGCAAAGATGTCAACGACTTGAAACAGTACAACAGTGATGAGCCAGAGCCC

AGATCACTAGCATGTTGGATCCAGAATGAATTCAACAAGGCGTGTGAACTGACTGACTCA

AGCTGGGTAGAACTTGATGAAATAGGGGAAGATGTTGCCCCAATCGAACACATTGCAAGC

ATGAGACGGAACTATTTTACAGCAGAGGTGTCCCACTGCAGGGCTACTGAATATATAATG

AAGGGAGTGTACATAAATACAGCTTTGCTCAATGCATCTTGTGCAGCCATGGATGACTTT

CAACTGATTCCAATGATAAGTAAATGTAGAACCAAAGAAGGAAGACGGAAAACAAACCTG

TATGGATTCATTATAAAAGGAAGATCTCATTTGAGGAATGATACCGACGTGGTAAACTTT

GTAAGTATGGAATTTTCCCTTACCGACCCAAGGTTGGAACCACATAAATGGGAAAAGTAT

TGTGTTCTTGAAATAGGGGACATGCTCCTGCGAACTGCAGTAGGCCAAGTGTCAAGACCC

ATGTTTCTGTATGTGAGAACCAATGGGACCTCCAAGATCAAGATGAAATGGGGTATGGAA

ATGAGACGCTGCCTTCTTCAATCTCTCCAACAGATTGAGAGCATGATTGAAGCTGAATCC

TCCGTCAAAGAGAAAGACCTGACCAAAGAATTCTTTGAAAACAAATCAGAAACATGGCCA

ATTGGAGAGTCACCTAAAGGAGTGGAGGAAGGTTCCATCGGGAAGGTGTGCAGAACCTTA

CTAGCAAAATCTGTATTCAACAGCCTATATGCATCTCCGCAACTCGAGGGGTTCTCAGCT

GAATCGAGAAAACTGCTACTCATTGTTCAGGCGCTTAGGGATAACCTGGAACCTGGAACC

TTTGATCTTGAAGGGCTATATGAAGCAATCGAGGAGTGCCTGATTAATGATCCCTGGGTT

TTGCTTAATGCATCTTGGTTCAACTCCTTCCTCACACATGCACTAAGATAG

>NP_2013consensus

ATGGCGTCTCAAGGCACCAAACGATCCTATGAACAGATGGAAACTGGTGGGGAACGCCAG

AATGCTACTGAGATCAGGGCATCTGTTGGAAGAATGGTTAGCGGCATTGGGAGATTCTAC

ATACAGATGTGTACAGAACTCAAACTCAGTGACAATGAAGGGAGGCTGATTCAGAACAGT

ATAACAATAGAGAGAATGGTACTCTCTGCATTTGATGAAAGAAGGAACAGATACCTGGAA

GAGCACCCCAGTGCAGGAAAGGACCCTAAGAAAACTGGAGGTCCAATTTACAGGAGAAGA

GACGGAAAATGGGTGAGAGAGCTGATCCTGTATGACAAAGAGGAAATCAGGAGAATTTGG

CGACAAGCGAACAATGGAGAGGATGCAACTGCTGGTCTTACCCATCTGATGATATGGCAT

TCCAACCTGAATGATGCTACCTATCAGAGAACGAGAGCTCTCGTGCGTACTGGAATGGAT

CCCCGGATGTGCTCTCTGATGCAAGGATCAACTCTCCCGAGGAGATCTGGAGCTGCAGGT

GCAGCAGTGAAGGGGATAGGGACAATGGTGATGGAACTGATTCGGATGATAAAACGAGGG

ATCAACGACCGGAATTTCTGGAGAGGCGAAAATGGAAGAAGGACAAGAATTGCATATGAG

AGAATGTGCAACATCCTCAAAGGGAAATTCCAAACAGCAGCACAAAGGGCAATGATGGAT

CAAGTGCGAGAGAGCAGAAATCCTGGGAATGCTGAAATAGAAGATCTCATTTTTCTGGCA

AGGTCTGCACTCATCCTGAGAGGATCAGTGGCTCATAAATCCTGCTTGCCTGCTTGTGTG

TACGGACTTGCAGTGGCCAGTGGATATGACTTTGAGAGAGAAGGGTACTCCTTGGTTGGA

ATAGATCCTTTCCGTCTGCTTCAAAACAGCCAGGTCTTTAGTCTCATTAGACCAAATGAG

AACCCAGCACATAAGAGCCAACTAGTGTGGATGGCATGCCACTCTGCAGCGTTTGAGGAC

CTTAGGGTCTCAAGTTTCATTAGAGGGACAAGAATGGTCCCAAGAGGACAGCTATCCACT

AGAGGGGTTCAAATTGCTTCAAATGAGAACATGGAAGCAATGGACTCCAATACTCTTGAA

CTGAGAAGTAGATATTGGGCTATAAGAACCAGAAGCGGAGGGAACACCAACCAACAGAGG

GCATCTGCAGGACAGGTCAGCGTTCAACCCACTTTCTCAGTACAGAGAAACCTTCCTTTC

GAAAGAGCAACCATTATGGCAGCATTTACAGGAAATACTGAGGGTAGAACGTCTGACATG

AGGACTGAAATCATAAGAATGATGGAAAGTGCCAGACCAGAAGATGTGTCATTCCAGGGG

CGGGGAGTCTTCGAGCTCTCGGACGAAAAGGCAACGAACCCGATCGTGCCTTCCTTTGAC

ATGAATAATGAAGGATCTTATTTCTTCGGAGACAATGCAGAGGAGTATGACAATTGA

>NS_2013consensus

ATGGATTCCAATACTGTGTCAAGCTTCCAGGTAGACTGCTTTCTTTGGCATGTCCGCAAA

CGATTTGCAGACCAAGAAATGGGTGATGCCCCATTTCTAGACCGGCTTCGCCGAGATCAG

AAGTCCCTGAGAGGAAGAAGCAGCACTCTTGGTCTGGACATCAGAACTGCCACGCGTGAA

GGAAAGCATATAGTGGAGCGGATTTTAGAGGAAGAGTCAGATGAAGCATTTAAAATGAGT

ATTGCTTCAGTGCCAGCTCCACGCTATCTAACTGACATGACTCTTGAAGAAATGTCAAGA

GATTGGTTAATGCTCATTCCCAAACAGAAAATAACAGGGTCCCTATGCATTAGAATGGAC

CAAGCAATAGTGGACAAAAACATCACATTGAAAGCAAATTTCAGTGTGATTTTCAATCGG

CTTGAAGCCCTGATACTACTTAGAGCTTTTACGGAAGAAGGAGCAATTGTAGGCGAAATC

TCACCATTACCTTCTCTTCCAGGACATACTGACAAGGATGTCAAAAATGCAATTGAGATC

CTCATCGGAGGATTTGAATGGAATGATAACACAGTTCGAGTCTCTGAAACTCTACAGAGA

TTCGCTTGGAGAAGCAGCGATGAGGATGGGAGATCTCCACTCTCTACAAAGTAGAAACGG

GAAATGGAGAGAACAGTTAAGCCAGAAGTTCGAAGAAATAAGATGGTTGATTGAAGAAGT

ACGACATAGATTAAAAATTACGGAGAATAGCTTTGAGCAAATAACTTTTATGCAAGCCTT

ACAACTATTGCTTGAAGTGGAGCAAGAGATAAGAACTTTCTCGTTTCAGCTTATTTAA

>MP_2013consensus

ATGAGTCTTCTAACCGAGGTCGAAACGTACGTTCTCTCTATCATTCCATCAGGCCCCCTC

AAAGCCGAGATCGCACAGAGACTTGAGGATGTTTTTGCAGGGAAGAACGCAGATCTCGAG

GCTCTCATGGAGTGGATAAAGACAAGACCAATCCTGTCACCTCTGACTAAGGGGATTTTA

GGGTTTGTGTTCACGCTCACCGTGCCCAGTGAGCGAGGACTGCAGCGTAGACGGTTTGTC

CAAAACGCCCTAAATGGGAATGGAGACCCAAACAACATGGACAAGGCGGTTAAATTATAC

AAGAAACTGAAGAGGGAAATGACATTTCATGGAGCAAAGGAAGTTGCACTCAGTTACTCA

ACTGGTGCGCTTGCCAGCTGCATGGGTCTCATATACAACAGAATGGGGACTGTGACCGCA

GAAGGGGCTCTTGGACTAGTATGTGCCACTTGTGAGCAGATTGCTGACGCACAACATCGG

TCCCACAGGCAGATGGCGACTACTACTAACCCACTAATTAGGCATGAGAATAGAATGGTA

CTAGCCAGCACTACGGCTAAGGCTATGGAGCAGATGGCTGGATCAAGTGAACAGGCAGCG

GAAGCCATGGAAGTTGCAAGTCAGGCTAGGCAAATGGTGCAGGCTATGAGAACAGTTGGG

ACTCACCCTAACTCCAGTACAGGTCTAAAAGATGATCTTATTGAAAATTTGCAGGCCTAC

CAGAACCGGATGGGAGTGCAACTGCAGCGGTTCAAGTGAGCCTCTAGTCGTTGCAGCTAA

CATTATTGGGATATTGCACTTGATATTGTGGATTCTTGATCGTCTTTTCTTCAAATGCAT

TTATCGTCGTTTTAAATACGGTTTGAAAAGAGGGCCTTCTACGGAAGGAATGCCTGAGTC

TATGAGGGAAGAATATCGGCAGGAACAGCAGAATGCTGTGGATGTTGACGATGGTCATTT

TGTCAACATAGAGCTGAAGTAA

>HA_2013consensus

ATGAACACTCAAATCCTGGTATTCGCTCTGATTGCGATCATTCCAACAAATGCAGACAAA

ATCTGCCTCGGACATCATGCCGTGTCAAACGGAACCAAAGTAAACACATTAACTGAAAGA

GGAGTGGAAGTCGTCAATGCAACTGAAACAGTGGAACGAACAAACATCCCCAGGATCTGC

TCAAAAGGGAAAAGGACAGTTGACCTCGGTCAATGTGGACTCCTGGGGACAATCACTGGA

CCACCTCAATGTGACCAATTCCTAGAATTTTCAGCCGATTTAATTATTGAGAGGCGAGAA

GGAAGTGATGTCTGTTATCCTGGGAAATTCGTGAATGAAGAAGCTCTGAGGCAAATTCTC

AGAGAATCAGGCGGAATTGACAAGGAAGCAATGGGATTCACATACAGTGGAATAAGAACT

AATGGAGCAACCAGTGCATGTAGGAGATCAGGATCTTCATTCTATGCAGAAATGAAATGG

CTCCTGTCAAACACAGATAATGCTGCATTCCCGCAGATGACTAAGTCATATAAAAATACA

AGAAAAAGCCCAGCTCTAATAGTATGGGGGATCCATCATTCCGTATCAACTGCAGAGCAA

ACCAAGCTATATGGGAGTGGAAACAAACTGGTGACAGTTGGGAGTTCTAATTATCAACAA

TCTTTTGTACCGAGTCCAGGAGCGAGACCACAAGTTAATGGTCTATCTGGAAGAATTGAC

TTTCATTGGCTAATGCTAAATCCCAATGATACAGTCACTTTCAGTTTCAATGGGGCTTTC

ATAGCTCCAGACCGTGCAAGCTTCCTGAGAGGAAAATCTATGGGAATCCAGAGTGGAGTA

CAGGTTGATGCCAATTGTGAAGGGGACTGCTATCATAGTGGAGGGACAATAATAAGTAAC

TTGCCATTTCAGAACATAGATAGCAGGGCAGTTGGAAAATGTCCGAGATATGTTAAGCAA

AGGAGTCTGCTGCTAGCAACAGGGATGAAGAATGTTCCTGAGATTCCAAAGGGAAGAGGC

CTATTTGGTGCTATAGCGGGTTTCATTGAAAATGGATGGGAAGGCCTAATTGATGGTTGG

TATGGTTTCAGACACCAGAATGCACAGGGAGAGGGAACTGCTGCAGATTACAAAAGCACT

CAATCGGCAATTGATCAAATAACAGGAAAATTAAACCGGCTTATAGAAAAAACCAACCAA

CAATTTGAGTTGATAGACAATGAATTCAATGAGGTAGAGAAGCAAATCGGTAATGTGATA

AATTGGACCAGAGATTCTATAACAGAAGTGTGGTCATACAATGCTGAACTCTTGGTAGCA

ATGGAGAACCAGCATACAATTGATCTGGCTGATTCAGAAATGGACAAACTGTACGAACGA

GTGAAAAGACAGCTGAGAGAGAATGCTGAAGAAGATGGCACTGGTTGCTTTGAAATATTT

CACAAGTGTGATGATGACTGTATGGCCAGTATTAGAAATAACACCTATGATCACAGCAAA

TACAGGGAAGAGGCAATGCAAAATAGAATACAGATTGACCCAGTCAAACTAAGCAGCGGC

TACAAAGATGTGATACTTTGGTTTAGCTTCGGGGCATCATGTTTCATACTTCTAGCCATT

GTAATGGGCCTTGTCTTCATATGTGTAAAGAATGGAAACATGCGGTGCACTATTTGTATA

TAA

>NA_consensus

ATGAATCCAAATCAGAAGATTCTATGCACTTCAGCCACTGCTATCATAATAGGCGCAATC

GCAGTACTCATTGGAATAGCAAACCTAGGATTGAACATAGGACTGCATCTAAAACCGGGC

TGCAATTGCTCACACTCACAACCTGAAACAACCAACACAAGCCAAACAATAATAAACAAC

TATTATAATGAAACAAACATCACCAACATCCAAATGGAAGAGAGAACAAGCAGGAATTTC

AATAACTTAACTAAAGGGCTCTGTACTATAAATTCATGGCACATATATGGGAAAGACAAT

GCAGTAAGAATTGGAGAGAGCTCGGATGTTTTAGTCACAAGAGAACCCTATGTTTCATGC

GACCCAGATGAATGCAGGTTCTATGCTCTCAGCCAAGGAACAACAATCAGAGGGAAACAC

TCAAACGGAACAATACACGATAGGTCCCAGTATCGCGCCCTGATAAGCTGGCCACTATCA

TCACCGCCCACAGTGTACAACAGCAGGGTGGAATGCATTGGGTGGTCAAGTACTAGTTGC

CATGATGGCAAATCCAGGATGTCAATATGTATATCAGGACCAAACAACAATGCATCTGCA

GTAGTATGGTACAACAGAAGGCCTGTTGCAGAAATTAACACATGGGCCCGAAACATACTA

AGAACACAGGAATCTGAATGTGTATGCCACAACGGCGTATGCCCAGTAGTGTTCACCGAT

GGGTCTGCCACTGGACCTGCAGACACAAGAATATACTATTTTAAAGAGGGGAAAATATTG

AAATGGGAGTCTCTGACTGGAACTGCTAAGCATATTGAAGAATGCTCATGTTACGGGGAA

CGAACAGGAATTACCTGCACATGCAGGGACAATTGGCAGGGCTCAAATAGACCAGTGATT

CAGATAGACCCAGTAGCAATGACACACACTAGTCAATATATATGCAGTCCTGTTCTTACA

GACAATCCCCGACCGAATGACCCAAATATAGGTAAGTGTAATGACCCTTATCCAGGTAAT

AATAACAATGGAGTCAAGGGATTCTCATACCTGGATGGGGCTAACACTTGGCTAGGGAGG

ACAATAAGCACAGCCTCGAGGTCTGGATACGAGATGTTAAAAGTGCCAAATGCATTGACA

GATGATAGATCAAAGCCCATTCAAGGTCAGACAATTGTATTAAACGCTGACTGGAGTGGT

TACAGTGGATCTTTCATGGACTATTGGGCTGAAGGGGACTGCTATCGAGCGTGTTTTTAT

GTGGAGTTGATACGTGGAAGACCCAAGGAGGATAAAGTGTGGTGGACCAGCAATAGTATA

GTATCGATGTGTTCCAGTACAGAATTCCTGGGACAATGGAACTGGCCTGATGGGGCTAAA

ATAGAGTACTTCCTCTAA

**2013 H7N9 Human Consensus Sequences (Theoretical translations of consensus open reading frames, for comparisons in Table 5):**

PB2: MERIKELRDLMSQSRTREILTKTTVDHMAIIKKYTSGRQEKNPALRMKWMMAMKYPITADKRIMEMIPERNEQGQTLWSKTNDAGSDRVMVSPLAVTWWNRNGPTTSTVHYPKVYKTYFEKVERLKHGTFGPVHFRNQVKIRRRVDINPGHADLSAKEAQDVIMEVVFPNEVGARILTSESQLTITKEKKKELQDCKIAPLMVAYMLERELVRKTRFLPVAGGTSSVYIEVLHLTQGTCWEQMYTPGGEVRNDDVDQSLIIAARNIVRRATVSADPLASLLEMCHSTQIGGVRMVDILRQNPTEEQAVDICKAAMGLRISSSFSFGGFTFKRTSGSSVKREEEVLTGNLQTLKIRVHEGYEEFTMVGRRATAILRKATRRLIQLIVSGKDEQSIAEAIIVAMVFSQEDCMIKAVRGDLNFVNRANQRLNPMHQLLRHFQKDAKVLFQNWGIEPIDNVMGMIGILPDMTPSTEMSLRGVRVSKMGVDEYSSTERVVVSIDRFLRVRDQRGNVLLSPEEVSETQGTEKLTITYSSSMMWEINGPESVLVNTYQWIIRNWENVKIQWSQDPTMLYNKMEFEPFQSLVPKAARGQYSGFVRVLFQQMRDVLGTFDTVQIIKLLPFAAAPPKQSRMQFSSLTVNVRGSGMRIVVRGNSPVFNYNKATKRLTVLGKDAGALMEDPDEGTAGVESAVLRGFLILGKEDKRYGPALSINELSNLAKGEKANVLIGQGDVVLVMKRKRDSSILTDSQTATKRIRMAIN

PB1: MDVNPTLLFLKVPVQNAISTTFPYTGDPPYSHGTGTGYTMDTVNRTHKYSEKGKWTTNTETGAPQLNPIDGPLPEDNEPSGYAQTDCVLEAMAFLEESHPGIFENSCLETMEIVQQTRVDKLTQGRQTYDWTLNRNQPAATALANTIEVFRSNGLTANESGRLIDFLKDVMDSMDKEEMEITTHFQRKRRVRDNMTKKMVTQRTIGKKKQRLNKRSYLIRALTLNTMTKDAERGKLKRRAIATPGMQIRGFVYFVEALARSICEKLEQSGLPVGGNEKKAKLANVVRKMMTNSQDTELSFTITGDNTKWNENQNPRMFLAMITYITRNQPEWFRNVLSIAPIMFSNKMARLGKGYMFESKSMKLRTQVPAEMLANIDLKYFNKSTREKIEKIRPLLIDGTASLSPGMMMGMFNMLSTVLGVSILNLGQKKYTKTTYWWDGLQSSDDFALIVNAPNHEGIQAGVDRFYRTCKLVGINMSKKKSYINRTGTFEFTSFFYRYGFVANFSMELPSFGVSGINESADMSVGVTVIKNNMINNDLGPATAQMALQLFIKDYRYTYRCHRGDTQIQTRRAFELKKLWEQTRSKAGLLVSDGGPNLYNIRNLHIPEVCLKWELMDEDYQGRLCNPMNPFVSHKEIDSVNNAVVMPAHGPAKSMEYDAVATTHSWIPKRNRSILNTSQRGILEDEQMYQKCCNLFEKFFPSSSYRRPVGISSMVEAMVSRARIDARIDFESGRIKKEEFAEIMKICSTIEELRRQK

PB1F2: MEQEQDTPWTQSTEHINTQKKESGQRTQRLEHPNSIQLMDHYLRTTSRVGMHKRIVYWKQWLSLKNLTQGSLKTRVSKRWKLFSKQEWIN

PA: MEDFVRQCFNPMIVELAEKAMKEYGEDPKIETNKFASICTHLEVCFMYSDFHFIDERGESTIIESGDPNALLKHRFEIIEGRDRTMAWTVVNSICNTTGAEKPKFLPDLYDYKENRFIEIGVTRREVHIYYLEKANKIKSEKTHIHIFSFTGEEMATKADYTLDEESRARIKTRLFTIRQEMASRGLWDSFRQSERGEETIEERFEITGTMRRLADQSLPPNFSSLENFRAYVDGFEPNGCIEGKLSQMSKEVNARIEPFLRTTPRPLRLPNGPPCSQRSKFLLMDALKLSIEDPSHEGEGIPLYDAIKCMKTFFGWKEPNIIKPHEKGINPNYLLTWKQVLAELQDIENEEKIPRTKNMKKTSQLKWALGENMAPEKVDFEDCKDVNDLKQYNSDEPEPRSLACWIQNEFNKACELTDSSWVELDEIGEDVAPIEHIASMRRNYFTAEVSHCRATEYIMKGVYINTALLNASCAAMDDFQLIPMISKCRTKEGRRKTNLYGFIIKGRSHLRNDTDVVNFVSMEFSLTDPRLEPHKWEKYCVLEIGDMLLRTAVGQVSRPMFLYVRTNGTSKIKMKWGMEMRRCLLQSLQQIESMIEAESSVKEKDLTKEFFENKSETWPIGESPKGVEEGSIGKVCRTLLAKSVFNSLYASPQLEGFSAESRKLLLIVQALRDNLEPGTFDLEGLYEAIEECLINDPWVLLNASWFNSFLTHALR

PA-X: MEDFVRQCFNPMIVELAEKAMKEYGEDPKIETNKFASICTHLEVCFMYSDFHFIDERGESTIIESGDPNALLKHRFEIIEGRDRTMAWTVVNSICNTTGAEKPKFLPDLYDYKENRFIEIGVTRREVHIYYLEKANKIKSEKTHIHIFSFTGEEMATKADYTLDEESRARIKTRLFTIRQEMASRGLWDSFVSLKEAKKQLKKDLKSQEPCAGLPTKVSHRTSPALKTLEPMWMDSNRTAALRASFLRCQKK

HA: MNTQILVFALIAIIPTNADKICLGHHAVSNGTKVNTLTERGVEVVNATETVERTNIPRICSKGKRTVDLGQCGLLGTITGPPQCDQFLEFSADLIIERREGSDVCYPGKFVNEEALRQILRESGGIDKEAMGFTYSGIRTNGATSACRRSGSSFYAEMKWLLSNTDNAAFPQMTKSYKNTRKSPALIVWGIHHSVSTAEQTKLYGSGNKLVTVGSSNYQQSFVPSPGARPQVNGLSGRIDFHWLMLNPNDTVTFSFNGAFIAPDRASFLRGKSMGIQSGVQVDANCEGDCYHSGGTIISNLPFQNIDSRAVGKCPRYVKQRSLLLATGMKNVPEIPKGRGLFGAIAGFIENGWEGLIDGWYGFRHQNAQGEGTAADYKSTQSAIDQITGKLNRLIEKTNQQFELIDNEFNEVEKQIGNVINWTRDSITEVWSYNAELLVAMENQHTIDLADSEMDKLYERVKRQLRENAEEDGTGCFEIFHKCDDDCMASIRNNTYDHSKYREEAMQNRIQIDPVKLSSGYKDVILWFSFGASCFILLAIVMGLVFICVKNGNMRCTICI

NP: MASQGTKRSYEQMETGGERQNATEIRASVGRMVSGIGRFYIQMCTELKLSDNEGRLIQNSITIERMVLSAFDERRNRYLEEHPSAGKDPKKTGGPIYRRRDGKWVRELILYDKEEIRRIWRQANNGEDATAGLTHLMIWHSNLNDATYQRTRALVRTGMDPRMCSLMQGSTLPRRSGAAGAAVKGIGTMVMELIRMIKRGINDRNFWRGENGRRTRIAYERMCNILKGKFQTAAQRAMMDQVRESRNPGNAEIEDLIFLARSALILRGSVAHKSCLPACVYGLAVASGYDFEREGYSLVGIDPFRLLQNSQVFSLIRPNENPAHKSQLVWMACHSAAFEDLRVSSFIRGTRMVPRGQLSTRGVQIASNENMEAMDSNTLELRSRYWAIRTRSGGNTNQQRASAGQVSVQPTFSVQRNLPFERATIMAAFTGNTEGRTSDMRTEIIRMMESARPEDVSFQGRGVFELSDEKATNPIVPSFDMNNEGSYFFGDNAEEYDN

NA: MNPNQKILCTSATAIIIGAIAVLIGIANLGLNIGLHLKPGCNCSHSQPETTNTSQTIINNYYNETNITNIQMEERTSRNFNNLTKGLCTINSWHIYGKDNAVRIGESSDVLVTREPYVSCDPDECRFYALSQGTTIRGKHSNGTIHDRSQYRALISWPLSSPPTVYNSRVECIGWSSTSCHDGKSRMSICISGPNNNASAVVWYNRRPVAEINTWARNILRTQESECVCHNGVCPVVFTDGSATGPADTRIYYFKEGKILKWESLTGTAKHIEECSCYGERTGITCTCRDNWQGSNRPVIQIDPVAMTHTSQYICSPVLTDNPRPNDPNIGKCNDPYPGNNNNGVKGFSYLDGANTWLGRTISTASRSGYEMLKVPNALTDDRSKPIQGQTIVLNADWSGYSGSFMDYWAEGDCYRACFYVELIRGRPKEDKVWWTSNSIVSMCSSTEFLGQWNWPDGAKIEYFL

M1: MSLLTEVETYVLSIIPSGPLKAEIAQRLEDVFAGKNADLEALMEWIKTRPILSPLTKGILGFVFTLTVPSERGLQRRRFVQNALNGNGDPNNMDKAVKLYKKLKREMTFHGAKEVALSYSTGALASCMGLIYNRMGTVTAEGALGLVCATCEQIADAQHRSHRQMATTTNPLIRHENRMVLASTTAKAMEQMAGSSEQAAEAMEVASQARQMVQAMRTVGTHPNSSTGLKDDLIENLQAYQNRMGVQLQRFK

M2: MSLLTEVETPTRTGWECNCSGSSEPLVVAANIIGILHLILWILDRLFFKCIYRRFKYGLKRGPSTEGMPESMREEYRQEQQNAVDVDDGHFVNIELK

NS1: MDSNTVSSFQVDCFLWHVRKRFADQEMGDAPFLDRLRRDQKSLRGRSSTLGLDIRTATREGKHIVERILEEESDEAFKMSIASVPAPRYLTDMTLEEMSRDWLMLIPKQKITGSLCIRMDQAIVDKNITLKANFSVIFNRLEALILLRAFTEEGAIVGEISPLPSLPGHTDKDVKNAIEILIGGFEWNDNTVRVSETLQRFAWRSSDEDGRSPLSTK

NS2/NEP: MDSNTVSSFQDILTRMSKMQLRSSSEDLNGMITQFESLKLYRDSLGEAAMRMGDLHSLQSRNGKWREQLSQKFEEIRWLIEEVRHRLKITENSFEQITFMQALQLLLEVEQEIRTFSFQLI

**2014 H7N9 Human Consensus Sequences (Theoretical translations of consensus open reading frames, for comparisons in Table 5):**

PB2:

MERIKELRDLMSQSRTREILTKTTVDHMAIIKKYTSGRQEKNPALRMKWMMAMKYPITADKRIMEMIPERNEQGQTLWSKTNDAGSDRVMVSPLAVTWWNRNGPTTSTVHYPKVYKTYFEKVERLKHGTFGPVHFRNQIKIRRRVDINPGHADLSAKEAQDVIMEVVFPNEVGARILTSESQLTITKEKKEELQDCKIAPLMVAYMLERELVRKTRFLPVAGGTSSVYIEVLHLTQGTCWEQMYTPGGEVRNDDVDQSLIIAARNIVRRATVSADPLASLLEMCHSTQIGGVRMVDILRQNPTEEQAVDICKAAMGLRISSSFSFGGFTFKRTSGSSVKREEEVLTGNLQTLKIRVHEGYEEFTMVGRRATAILRKATRRLIQLIVSGKDEQSIAEAIIVAMVFSQEDCMIKAVRGDLNFVNRANQRLNPMHQLLRHFQKDAKVLFQNWGIEPIDNVMGMIGILPDMTPSTEMSLRGVRVSKMGVDEYSSTERVVVSIDRFLRVRDQRGNVLLSPEEVSETQGTEKLTITYSSSMMWEINGPESVLVNTYQWIIRNWETVKIQWSQDPTILYNKMEFEPFQSLVPKAARGQYSGFVRVLFQQMRDVLGTFDTVQIIKLLPFAAAPPKQSRMQFSSLTVNVRGSGMRIVVRGNSPVFNYNKATKRLTVLGKDAGALVEDPDEGTAGVESAVLRGFLILGKEDKRYGPALSINELSNLAKGEKANVLIGQGDVVLVMKRKRDSSILTDSQTATKRIRMAIN

PB1:

MDVNPTLLFLKVPVQNAISTTFPYTGDPPYSHGTGTGYTMDTVNRTHKYSEKGKWTTNTETGAPQLNPIDGPLPEDNEPSGYAQTDCVLEAMAFLEESHPGIFENSCLETMEIVQQTRVDKLTQGRQTYDWTLNRNQPAATALANTIEVFRSNGLTANESGRLIDFLKDVVDSMDKEEMEITTHFQRKRRVRDNMTKKMVTQRTIGKKKQRLNKRSYLIRALTLNTMTKDAERGKLKRRAIATPGMQIRGFVYFVEALARSICEKLEQSGLPVGGNEKKAKLANVVRKMMTNSQDTELSFTITGDNTKWNENQNPRMFLAMITYITRNQPEWFRNVLSIAPIMFSNKMARLGKGYMFESKSMKLRTQVPAEMLANIDLKYFNKSTREKIEKIRPLLMDGTASLSPGMMMGMFNMLSTVLGVSILNLGQKKYTKTTYWWDGLQSSDDFALIVNAPNHEGIQAGVDRFYRTCKLVGINMSKKKSYINRTGTFEFTSFFYRYGFVANFSMELPSFGVSGINESADMSIGVTVIKNNMINNDLGPATAQMALQLFIKDYRYTYRCHRGDTQIQTRRAFELKKLWEQTRSKAGLLVSDGGPNLYNIRNLHIPEVCLKWELMDEDYQGRLCNPMNPFVSHKEIDSVNNAVVMPAHGPAKSMEYDAVATTHSWIPKRNRSILNTSQRGILEDEQMYQKCCXLFEKFFPSSSYRRPVGISSMVEAMVSRARIDARIDFESGRIKKEEFAEIMKICSTIEELRRQK

PB1F2:

MEQEQDTPWTQSTEHINTQKKESGQRTQRLEHPNSIRLMDRCLRTTSRVGMHKRIVYWRQWLSLKNLIQGFLKTRVSKRWKLFSKQEWIN

PA:

MEDFVRQCFNPMIVELAEKAMKEYGEDPKIETNKFASICTHLEVCFMYSDFHFIDERGESTIIESGDPNALLKHRFEIIEGRDRTMAWTVVNSICNTTGAEKPKFLPDLYDYKENRFIEIGVTRREVHIYYLEKANKIKSEKTHIHIFSFTGEEMATKADYTLDEESRARIKTRLFTIRQEMASRGLWDSFRQSERGEETIEERFEITGTMRRLADQSLPPNFSSLENFRAYVDGFEPNGCIEGKLSQMSKEVNARIEPFLRTTPRPLRLPNGPPCSQRSKFLLMDALKLSIEDPSHEGEGIPLYDAIKCMKTFFGWKEPNIIKPHEKGINPNYLLTWKQVLAELQDIENEEKIPRTKNMKKTSQLKWALGENMAPEKVDFEDCKDVNDLKQYNSDEPEPRSLACWIQNEFNKACELTDSSWVELDEIGEDVAPIEHIASMRRNYFTAEVSHCRATEYIMKGVYINTALLNASCAAMDDFQLIPMISKCRTKEGRRKTNLYGFIIKGRSHLRNDTDVVNFVSMEFSLTDPRLEPHKWEKYCVLEIGDMLLRTAVGQVSRPMFLYVRTNGTSKIKMKWGMEMRRCLLQSLQQIESMIEAESSVKEKDLTKEFFENKSETWPIGESPKGVEEGSIGKVCRTLLAKSVFNSLYASPQLEGFSAESRKLLLIVQALRDNLEPGTFDLEGLYEAIEECLINDPWVLLNASWFNSFLTHALR

PA-X:

MEDFVRQCFNPMIVELAEKAMKEYGEDPKIETNKFASICTHLEVCFMYSDFHFIDERGESTIIESGDPNALLKHRFEIIEGRDRTMAWTVVNSICNTTGAEKPKFLPDLYDYKENRFIEIGVTRREVHIYYLEKANKIKSEKTHIHIFSFTGEEMATKADYTLDEESRARIKTRLFTIRQEMASRGLWDSFVSPKEAKKQLKKDLKSQEPCAGLPTKVSHRTSPALKTLEPMWMDSNRTAALRASFLRCQKK

HA:

MNTQILVFALIAIIPTNADKICLGHHAVSNGTKVNTLTERGVEVVNATETVERTNIPRICSKGKKTVDLGQCGLLGTITGPPQCDQFLEFSADLIIERREGSDVCYPGKFVNEEALRQILRESGGIDKEAMGFTYSGIRTNGATSACRRSGSSFYAEMKWLLSNTDNAAFPQMTKSYKNTRKSPALIVWGIHHSVSTAEQTKLYGSGNKLVTVGSSNYQQSFVPSPGARPQVNGLSGRIDFHWLMLNPNDTVTFSFNGAFIAPDRASFLRGKSMGIQSGVQVDANCEGDCYHSGGTIISNLPFQNIDSRAVGKCPRYVKQRSLLLATGMKNVPEIPKGRGLFGAIAGFIENGWEGLIDGWYGFRHQNAQGEGTAADYKSTQSAIDQITGKLNRLIEKTNQQFELIDNEFNEVEKQIGNVINWTRDSITEVWSYNAELLVAMENQHTIDLADSEMDKLYERVKRQLRENAEEDGTGCFEIFHKCDDDCMASIRNNTYDHSKYREEAMQNRIQIDPVKLSSGYKDVILWFSFGASCFILLAIVMGLVFICVKNGNMRCTICI

NP:

MASQGTKRSYEQMETGGERQNATEIRASVGRMVSGIGRFYIQMCTELKLSDNEGRLIQNSITIERMVLSAFDERRNRYLEEHPSAGKDPKKTGGPIYRRRDGKWVRELILYDKEEIRRIWRQANNGEDATAGLTHLMIWHSNLNDATYQRTRALVRTGMDPRMCSLMQGSTLPRRSGAAGAAVKGIGTMVMELIRMIKRGINDRNFWRGENGRRTRIAYERMCNILKGKFQTAAQRAMMDQVRESRNPGNAEIEDLIFLARSALILRGSVAHKSCLPACVYGLAVASGYDFEREGYSLVGIDPFRLLQNSQVFSLIRPNENPAHKSQLVWMACHSAAFEDLRVSSFIRGTRMVPRGQLSTRGVQIASNENIEAMESNTLELRSRYWAIRTRSGGNTNQQRASAGQVSVQPTFSVQRNLPFERATIMAAFTGNTEGRTSDMRTEIIRMMESARPEDVSFQGRGVFELSDEKATNPIVPSFDMNNEGSYFFGDNAEEYDN

NA:

MNPNQKILCTSATAIIIGAIAVLIGIANLGLNIGLHLKPGCNCSHSQPETTNTSQTIINNYYNETNITNIQMEERTSRNFNNLTKGLCTINSWHIYGKDNAVRIGESSDVLVTREPYVSCDPDECRFYALSQGTTIRGKHSNGTIHDRSQYRALISWPLSSPPTVYNSRVECIGWSSTSCHDGKSRMSICISGPNNNASAVVWYNRRPVAEINTWARNILRTQESECVCHNGVCPVVFTDGSATGPADTRIYYFKEGKILKWESLTGTAKHIEECSCYGERTGITCTCRDNWQGSNRPVIQIDPVAMTHTSQYICSPVLTDNPRPNDPNIGKCNDPYPGNNNNGVKGFSYLDGANTWLGRTISTASRSGYEMLKVPNALTDDRSKPIQGQTIVLNADWSGYSGSFMDYWAEGDCYRACFYVELIRGRPKEDKVWWTSNSIVSMCSSTEFLGQWNWPDGAKIEYFL

M1:

MSLLTEVETYVLSIIPSGPLKAEIAQRLEDVFAGKNADLEALMEWIKTRPILSPLTKGILGFVFTLTVPSERGLQRRRFVQNALNGNGDPNNMDKAVKLYKKLKREMTFHGAKEVALSYSTGALASCMGLIYNRMGTVTAEGALGLVCATCEQIADAQHRSHRQMATTTNPLIRHENRMVLASTTAKAMEQMAGSSEQAAEAMEVASQARQMVQAMRTVGTHPNSSTGLKDDLIENLQAYQNRMGVQLQRFK

M2:

MSLLTEVETPTRTGWECNCSGSSEPLVVAANIIGILHLILWILDRLFFKCIYRRFKYGLKRGPSTEGMPESMREEYRQEQQNAVDVDDGHFVNIELK

NS1:

MDSNTVSSFQVDCFLWHVRKRFADQELGDAPFLDRLRRDQKSLRGRSSTLGLDIRTATREGKHIVERILEEESDEAFKMTIASVPAPRYLTDMTLEEMSRDWLMLIPKQKVTGSLCIRMDQAIVDKNITLKANFSVIFNRLEALILLRAFTDEGAIVGEISPLPSLPGHTDKDVKNAIEILIGGFEWNDNTVRVSETLQRFAWRSSDEDGRPPLSPK

NS2/NEP:

MDSNTVSSFQDILTRMSKMQLRSSSEDLNGMITQFESLKLYRDSLGEAAMRMGDLHSLQSRNGKWREQLSQKFEEIRWLIEEVRHRLKITENSFEQITFMQALQLLLEVEQEIRTFSFQLI

**2015 H7N9 Human Consensus Sequences (Theoretical translations of consensus open reading frames, for comparisons in Table 5):**

PB2:

MERIKELRDLMSQSRTREILTKTTVDHMAIIKKYTSGRQEKNPALRMKWMMAMKYPITADKRIMEMIPERNEQGQTLWSKTNDAGSDRVMVSPLAVTWWNRNGPTTSTVHYPKVYKTYFEKVERLKHGTFGPVHFRNQVKIRRRVDINPGHADLSAKEAQDVIMEVVFPNEVGARILTSESQLTITKEKKEELQDCKIAPLMVAYMLERELVRKTRFLPVAGGTSSVYIEVLHLTQGTCWEQMYTPGGEVRNDDVDQSLIIAARNIVRRATVSADPLASLLEMCHSTQIGGVRMVDILRQNPTEEQAVDICKAAMGLRISSSFSFGGFTFKRTSGSSVKREEEVLTGNLQTLKIRVHEGYEEFTMVGRRATAILRKATRRLIQLIVSGKDEQSIAEAIIVAMVFSQEDCMIKAVRGDLNFVNRANQRLNPMHQLLRHFQKDAKVLFQNWGIEPIDNVMGMIGILPDMTPSTEMSLRGVRVSKMGVDEYSSTERVVVSIDRFLRVRDQRGNILLSPEEVSETQGTEKLTITYSSSLMWEINGPESVLVNTYQWIIRNWETVKIQWSQDPTILYNKMEFEPFQSLVPKAARGQYSGFVRVLFQQMRDVLGTFDTVQIIKLLPFAAAPPKQSRMQFSSLTVNVRGSGMRVVVRGNSPVFNYNKATKRLTVLGKDAGALMEDPDEGTAGVESAVLRGFLILGKEDKRYGPALSINELSNLAKGEKANVLIGQGDVVLVMKRKRDSSILTDSQTATKRIRMAIN

PB1:

MDVNPTLLFLKVPVQNAISTTFPYTGDPPYSHGTGTGYTMDTVNRTHKYSEKGKWTTNTETGAPQLNPIDGPLPEDNEPSGYAQTDCVLEAMAFLEESHPGIFENSCLETMEIVQQTRVDKLTQGRQTYDWTLNRNQPAATALANTIEVFRSNGLTANESGRLIDFLKDVMDSMDKEEMEITTHFQRKRRVRDNMTKKMVTQRTIGKKKQRLNKRSYLIRALTLNTMTKDAERGKLKRRAIATPGMQIRGFVYFVEALARSICEKLEQSGLPVGGNEKKAKLANVVRKMMTNSQDTELSFTITGDNTKWNENQNPRMFLAMITYITRNQPEWFRNVLSIAPIMFSNKMARLGKGYMFESKSMKLRTQVPAEMLANIDLKYFNKSTREKIEKIRPLLIDGTASLSPGMMMGMFNMLSTVLGVSILNLGQKKYTKTTYWWDGLQSSDDFALIVNAPNHEGIQAGVDRFYRTCKLVGINMSKKKSYINRTGTFEFTSFFYRYGFVANFSMELPSFGVSGINESADMSVGVTVIKNNMINNDLGPATAQMALQLFIKDYRYTYRCHRGDTQIQTRRAFELKKLWEQTRSKAGLLVSDGGPNLYNIRNLHIPEVCLKWELMDEDYQGRLCNPMNPFVSHKEIDSVNNAVVMPAHGPAKSMEYDAVATTHSWIPKRNRSILNTSQRGILEDEQMYQKCCNLFEKFFPSSSYRRPVGISSMVEAMVSRARIDARIDFESGRIKKEEFAEIMKICSTIEELRRQK

PB1F2:

MEQEQDTPWTQSTEHINTQKKESGQRTQRLEHPNSIQLMDHYLRTTSRVGMHKRIVYWKQWLSLKNLTQGSLKTRVSKRWKLFSKQEWIN

PA: MEDFVRQCFNPMIVELAEKAMKEYGEDPKIETNKFASICTHLEVCFMYSDFHFIDERGESTIIESGDPNALLKHRFEIIEGRDRTMAWTVVNSICNTTGAEKPKFLPDLYDYKENRFIEIGVTRREVHIYYLEKANKIKSEKTHIHIFSFTGEEMATKADYTLDEESRARIKTRLFTIRQEMASRGLWDSFRQSERGEETIEERFEITGTMRRLADQSLPPNFSSLENFRAYVDGFEPNGCIEGKLSQMSKEVNARIEPFLRTTPRPLRLPNGPPCSQRSKFLLMDALKLSIEDPSHEGEGIPLYDAIKCMKTFFGWKEPNIIKPHEKGINPNYLLTWKQVLAELQDIENEEKIPRTKNMKKTSQLKWALGENMAPEKVDFEDCKDVNDLKQYNSDEPEPRSLACWIQNEFNKACELTDSSWVELDEIGEDVAPIEHIASMRRNYFTAEVSHCRATEYIMKGVYINTALLNASCAAMDDFQLIPMISKCRTKEGRRKTNLYGFIIKGRSHLRNDTDVVNFVSMEFSLTDPRLEPHKWEKYCVLEIGDMLLRTAVGQVSRPMFLYVRTNGTSKIKMKWGMEMRRCLLQSLQQIESMIEAESSVKEKDLTKEFFENKSETWPIGESPKGVEEGSIGKVCRTLLAKSVFNSLYASPQLEGFSAESRKLLLIVQALRDNLEPGTFDLEGLYEAIEECLINDPWVLLNASWFNSFLTHALR

PA-X:

MEDFVRQCFNPMIVELAEKAMKEYGEDPKIETNKFASICTHLEVCFMYSDFHFIDERGESTIIESGDPNALLKHRFEIIEGRDRTMAWTVVNSICNTTGAEKPKFLPDLYDYKENRFIEIGVTRREVHIYYLEKANKIKSEXTHIHIFSFTGEEMATKADYTLDEESRARIKTRLFTIRQEMASRGLWDSFVSPKEAKKQLKKDLKSQEPCAGLPTKVSHRTSPALKTLEPMWMDSNRTAALRASFLRCQKK

HA:

MNTQILVFALIAIIPTNADKICLGHHAVSNGTKVNTLTERGVEVVNATETVERTNIPRICSKGKRTVDLGQCGLLGTITGPPQCDQFLEFSADLIIERREGSDVCYPGKFVNEEALRQILRESGGIDKEAMGFTYNGIRTNGVTSACRRSGSSFYAEMKWLLSNTDNAAFPQMTKSYKNTRKSPAIIVWGIHHSVSTAEQTKLYGSGNKLVTVGSSNYQQSFVPSPGARPQVNGLSGRIDFHWLMLNPNDTVTFSFNGAFIAPDRASFLRGKSMGIQSGVQVDANCEGDCYHSGGTIISNLPFQNIDSRAVGKCPRYVKQRSLLLATGMKNVPEIPKGRGLFGAIAGFIENGWEGLIDGWYGFRHQNAQGEGTAADYKSTQSAIDQITGKLNRLIAKTNQQFELIDNEFNEVEKQIGNVINWTRDSITEVWSYNAELLVAMENQHTIDLADSEMDKLYERVKRQLRENAEEDGTGCFEIFHKCDDDCMASIRNNTYDHRKYREEAMQNRIQIDPVKLSSGYKDVILWFSFGASCFILLAIVMGLVFICVKNGNMRCTICI

NP:

MASQGTKRSYEQMETGGERQNATEIRASVGRMVSGIGRFYIQMCTELKLSDNEGRLIQNSITIERMVLSAFDERRNRYLEEHPSAGKDPKKTGGPIYRRRDGKWVRELILYDKEEIRRIWRQANNGEDATAGLTHLMIWHSNLNDATYQRTRALVRTGMDPRMCSLMQGSTLPRRSGAAGAAVKGIGTMVMELIRMIKRGINDRNFWRGENGRRTRIAYERMCNILKGKFQTAAQRAMMDQVRESRNPGNAEIEDLIFLARSALILRGSVAHKSCLPACVYGLAVASGYDFEREGYSLVGIDPFRLLQNSQVFSLIRPNENPAHKSQLVWMACHSAAFEDLRVSSFIRGTRMVPRGQLSTRGVQIASNENMEAMDSNTLELRSRYWAIRTRSGGNTNQQRASAGQVSVQPTFSVQRNLPFERATIMAAFTGNTEGRTSDMRTEIIRMMESARPEDVSFQGRGVFELSDEKATNPIVPSFDMNNEGSYFFGDNAEEYDN

NA:

MNPNQKILCTSATAITIGAIAVLIGIANLGLNIGLHLKPGCNCSHSQPETTNTSQTIINNYYNETNITNIQMEERTSRNFNNLTKGLCTINSWHIYGKDNAVRIGESSDVLVTREPYVSCDPDECRFYALSQGTTIRGKHSNGTIHDRSQYRALISWPLSSPPTVYNSRVECIGWSSTSCHDGKSRMSICISGPNNNASAVVWYNRRPVAEINTWARNILRTQESECVCHNGVCPVVFTDGPATGPADTRIYYFKEGKILKWESLTGTAKHIEECSCYGERTGITCTCRDNWQGSNRPVIQIDPVAMTHTSQYICSPVLTDSPRPNDPNIGKCNDPYPGNNNNGVKGFSYLDGANTWLGRTISTASRSGYEMLKVPNALTDDRSKPIQGQTIVLNADWSGYSGSFMDYWAEGDCYRACFYVELIRGRPKEDKVWWTSNSIVSMCSSTEFLGQWNWPDGAKIEYFL

M1:

MSLLTEVETYVLSIIPSGPLKAEIAQRLEDVFAGKNADLEALMEWIKTRPILSPLTKGILGFVFTLTVPSERGLQRRRFVQNALNGNGDPNNMDKAVKLYKKLKREMTFHGAKEVALSYSTGALASCMGLIYNRMGTVTAEGALGLVCATCEQIADAQHRSHRQMATTTNPLIRHENRMVLASTTAKAMEQMAGSSEQAAEAMEVASQARQMVQAMRTVGTHPNSSTGLKDDLIENLQAYQNRMGVQLQRFK

M2:

MSLLTEVETLTRTGWECNCSGSSDPLVVAANIIGILHLILWILDRLFFKCIYRRFKYGLKRGPSTEGMPESMREEYRQEQQNAVDVDDGHFVNIELK

NS1:

MDSNTVSSFQVDCFLWHVRKRFADQEMGDAPFLDRLRRDQKSLRGRSSTLGLDIRTATREGKHIVERILEEESDEAFKMSIASVPAPRYLTDMTLEEMSRDWLMLIPKQKITGSLCIRMDQAIVDKNITLKANFSVIFNRLEALILLRAFTEEGAIVGEISPLPSLPGHTDKDVKNAIEILIGGFEWNDNTVRVSETLQRFAWRSSDEDGRSPLSTK

NS2/NEP:

MDSNTVSSFQDILTRMSKMQLRSSSEDLNGMITQFESLKLYRDSLGEAAMRMGDLHSLQSRNGKWREQLSQKFEEIRWLIEEVRHRLKITENSFEQITFMQALQLLLEVEQEIRTFSFQLI

**2013 H7N9 Avian Consensus Sequences (Theoretical translations of consensus open reading frames, for comparisons in Table 5):**

PB2: MERIKELRDLMSQSRTREILTKTTVDHMAIIKKYTSGRQEKNPALRMKWMMAMKYPITADKRIMEMIPERNEQGQTLWSKTNDAGSDRVMVSPLAVTWWNRNGPTTSTVHYPKVYKTYFEKVERLKHGTFGPVHFRNQVKIRRRVDINPGHADLSAKEAQDVIMEVVFPNEVGARILTSESQLTITKEKKEELQDCKIAPLMVAYMLERELVRKTRFLPVAGGTSSVYIEVLHLTQGTCWEQMYTPGGEVRNDDVDQSLIIAARNIVRRATVSADPLASLLEMCHSTQIGGVRMVDILRQNPTEEQAVDICKAAMGLRISSSFSFGGFTFKRTSGSSVKREEEVLTGNLQTLKIRVHEGYEEFTMVGRRATAILRKATRRLIQLIVSGKDEQSIAEAIIVAMVFSQEDCMIKAVRGDLNFVNRANQRLNPMHQLLRHFQKDAKVLFQNWGIEPIDNVMGMIGILPDMTPSTEMSLRGVRVSKMGVDEYSSTERVVVSIDRFLRVRDQRGNVLLSPEEVSETQGTEKLTITYSSSMMWEINGPESVLVNTYQWIIRNWETVKIQWSQDPTMLYNKMEFEPFQSLVPKAARGQYSGFVRVLFQQMRDVLGTFDTVQIIKLLPFAAAPPEQSRMQFSSLTVNVRGSGMRIVVRGNSPVFNYNKATKRLTVLGKDAGALMEDPDEGTAGVESAVLRGFLILGKEDKRYGPALSINELSNLAKGEKANVLIGQGDVVLVMKRKRDSSILTDSQTATKRIRMAIN

PB1: MDVNPTLLFLKVPVQNAISTTFPYTGDPPYSHGTGTGYTMDTVNRTHKYSEKGKWTTNTETGAPQLNPIDGPLPEDNEPSGYAQTDCVLEAMAFLEESHPGIFENSCLETMEIVQQTRVDKLTQGRQTYDWTLNRNQPAATALANTIEVFRSNGLTANESGRLIDFLKDVMDSMDKEEMEITTHFQRKRRVRDNMTKKMVTQRTIGKKKQRLNKRSYLIRALTLNTMTKDAERGKLKRRAIATPGMQIRGFVYFVEALARSICEKLEQSGLPVGGNEKKAKLANVVRKMMTNSQDTELSFTITGDNTKWNENQNPRMFLAMITYITRNQPEWFRNVLSIAPIMFSNKMARLGKGYMFESKSMKLRTQVPAEMLANIDLKYFNKSTREKIEKIRPLLIDGTASLSPGMMMGMFNMLSTVLGVSILNLGQKKYTKTTYWWDGLQSSDDFALIVNAPNHEGIQAGVDRFYRTCKLVGINMSKKKSYINRTGTFEFTSFFYRYGFVANFSMELPSFGVSGINESADMSIGVTVIKNNMINNDLGPATAQMALQLFIKDYRYTYRCHRGDTQIQTRRAFELKKLWEQTRSKAGLLVSDGGPNLYNIRNLHIPEVCLKWELMDEDYQGRLCNPMNPFVSHKEIDSVNNAVVMPAHGPAKSMEYDAVATTHSWIPKRNRSILNTSQRGILEDEQMYQKCCNLFEKFFPSSSYRRPVGISSMVEAMVSRARIDARIDFESGRIKKEEFAEIMKICSTIEELRRQKX

PB1F2: MEQEQDTPWTQSTEHINTQKKESGQRTQRLEHPNSIQLMDHYLRTTSQVDMHKRIVYWKQWLSLKNLTQGSLKTRVSKRWKLFSKQEWTN

PA: MEDFVRQCFNPMIVELAEKAMKEYGEDPKIETNKFASICTHLEVCFMYSDFHFIDERGESTIIESGDPNALLKHRFEIIEGRDRTMAWTVVNSICNTTGAEKPKFLPDLYDYKENRFIEIGVTRREVHIYYLEKANKIKSEKTHIHIFSFTGEEMATKADYTLDEESRARIKTRLFTIRQEMASRGLWDSFRQSERGEETIEERFEITGTMRRLADQSLPPNFSSLENFRAYVDGFEPNGCIEGKLSQMSKEVNARIEPFLRTTPRPLRLPNGPPCSQRSKFLLMDALKLSIEDPSHEGEGIPLYDAIKCMKTFFGWKEPNIIKPHEKGINPNYLLTWKQVLAELQDIENEEKIPRTKNMKKTSQLKWALGENMAPEKVDFEDCKDVNDLKQYNSDEPEPRSLACWIQNEFNKACELTDSSWVELDEIGEDVAPIEHIASMRRNYFTAEVSHCRATEYIMKGVYINTALLNASCAAMDDFQLIPMISKCRTKEGRRKTNLYGFIIKGRSHLRNDTDVVNFVSMEFSLTDPRLEPHKWEKYCVLEIGDMLLRTAVGQVSRPMFLYVRTNGTSKIKMKWGMEMRRCLLQSLQQIESMIEAESSVKEKDLTKEFFENKSETWPIGESPKGVEEGSIGKVCRTLLAKSVFNSLYASPQLEGFSAESRKLLLIVQALRDNLEPGTFDLEGLYEAIEECLINDPWVLLNASWFNSFLTHALR

PA-X: MEDFVRQCFNPMIVELAEKAMKEYGEDPKIETNKFASICTHLEVCFMYSDFHFIDERGESTIIESGDPNALLKHRFEIIEGRDRTMAWTVVNSICNTTGAEKPKFLPDLYDYKENRFIEIGVTRREVHIYYLEKANKIKSEKTHIHIFSFTGEEMATKADYTLDEESRARIKTRLFTIRQEMASRGLWDSFVSLKEAKKQLKKDLKSQEPCAGLPTKVSHRTSPALKTLEPMWMDSNRTAALRASFLRCQKK

HA: MNTQILVFALIAIIPTNADKICLGHHAVSNGTKVNTLTERGVEVVNATETVERTNIPRICSKGKRTVDLGQCGLLGTITGPPQCDQFLEFSADLIIERREGSDVCYPGKFVNEEALRQILRESGGIDKEAMGFTYSGIRTNGATSACRRSGSSFYAEMKWLLSNTDNAAFPQMTKSYKNTRKSPALIVWGIHHSVSTAEQTKLYGSGNKLVTVGSSNYQQSFVPSPGARPQVNGLSGRIDFHWLMLNPNDTVTFSFNGAFIAPDRASFLRGKSMGIQSGVQVDANCEGDCYHSGGTIISNLPFQNIDSRAVGKCPRYVKQRSLLLATGMKNVPEIPKGRGLFGAIAGFIENGWEGLIDGWYGFRHQNAQGEGTAADYKSTQSAIDQITGKLNRLIEKTNQQFELIDNEFNEVEKQIGNVINWTRDSITEVWSYNAELLVAMENQHTIDLADSEMDKLYERVKRQLRENAEEDGTGCFEIFHKCDDDCMASIRNNTYDHSKYREEAMQNRIQIDPVKLSSGYKDVILWFSFGASCFILLAIVMGLVFICVKNGNMRCTICI

NP: MASQGTKRSYEQMETGGERQNATEIRASVGRMVSGIGRFYIQMCTELKLSDNEGRLIQNSITIERMVLSAFDERRNRYLEEHPSAGKDPKKTGGPIYRRRDGKWVRELILYDKEEIRRIWRQANNGEDATAGLTHLMIWHSNLNDATYQRTRALVRTGMDPRMCSLMQGSTLPRRSGAAGAAVKGIGTMVMELIRMIKRGINDRNFWRGENGRRTRIAYERMCNILKGKFQTAAQRAMMDQVRESRNPGNAEIEDLIFLARSALILRGSVAHKSCLPACVYGLAVASGYDFEREGYSLVGIDPFRLLQNSQVFSLIRPNENPAHKSQLVWMACHSAAFEDLRVSSFIRGTRMVPRGQLSTRGVQIASNENIEAMDSNTLELRSRYWAIRTRSGGNTNQQRASAGQVSVQPTFSVQRNLPFERATIMAAFTGNTEGRTSDMRTEIIRMMESARPEDVSFQGRGVFELSDEKATNPIVPSFDMNNEGSYFFGDNAEEYDN

NA: MNPNQKILCTSATAIIIGAIAVLIGIANLGLNIGLHLKPGCNCSHSQPETTNTSQTIINNYYNETNITNIQMEERTSRNFNNLTKGLCTINSWHIYGKDNAVRIGESSDVLVTREPYVSCDPDECRFYALSQGTTIRGKHSNGTIHDRSQYRALISWPLSSPPTVYNSRVECIGWSSTSCHDGKSRMSICISGPNNNASAVVWYNRRPVAEINTWARNILRTQESECVCHNGVCPVVFTDGSATGPADTRIYYFKEGKILKWESLTGTAKHIEECSCYGERTGITCTCRDNWQGSNRPVIQIDPVAMTHTSQYICSPVLTDNPRPNDPNIGKCNDPYPGNNNNGVKGFSYLDGANTWLGRTISTASRSGYEMLKVPNALTDDRSKPIQGQTIVLNADWSGYSGSFMDYWAEGDCYRACFYVELIRGRPKEDKVWWTSNSIVSMCSSTEFLGQWNWPDGAKIEYFL

M1: MSLLTEVETYVLSIIPSGPLKAEIAQRLEDVFAGKNADLEALMEWIKTRPILSPLTKGILGFVFTLTVPSERGLQRRRFVQNALNGNGDPNNMDKAVKLYKKLKREMTFHGAKEVALSYSTGALASCMGLIYNRMGTVTAEGALGLVCATCEQIADAQHRSHRQMATTTNPLIRHENRMVLASTTAKAMEQMAGSSEQAAEAMEVASQARQMVQAMRTVGTHPNSSTGLKDDLIENLQAYQNRMGVQLQRFK

M2: MSLLTEVETPTRTGWECNCSGSSEPLVVAANIIGILHLILWILDRLFFKCIYRRFKYGLKRGPSTEGMPESMREEYRQEQQNAVDVDDGHFVNIELK

NS1: MDSNTVSSFQVDCFLWHVRKRFADQEMGDAPFLDRLRRDQKSLRGRSSTLGLDIRTATREGKHIVERILEEESDEAFKMSIASVPAPRYLTDMTLEEMSRDWLMLIPKQKITGSLCIRMDQAIVDKNITLKANFSVIFNRLEALILLRAFTEEGAIVGEISPLPSLPGHTDKDVKNAIEILIGGFEWNDNTVRVSETLQRFAWRSSDEDGRSPLSTK

NS2/NEP: MDSNTVSSFQDILTRMSKMQLRSSSEDLNGMITQFESLKLYRDSLGEAAMRMGDLHSLQSRNGKWREQLSQKFEEIRWLIEEVRHRLKITENSFEQITFMQALQLLLEVEQEIRTFSFQLI

**2014 H7N9 Avian Consensus Sequences (Theoretical translations of consensus open reading frames, for comparisons in Table 5):**

PB2:

MERIKELRDLMSQSRTREILTKTTVDHMAIIKKYTSGRQEKNPALRMKWMMAMKYPITADKRIMEMIPERNEQGQTLWSKTNDAGSDRVMVSPLAVTWWNRNGPTTSTVHYPKVYKTYFEKVERLKHGTFGPVHFRNQVKIRRRVDINPGHADLSAKEAQDVIMEVVFPNEVGARILTSESQLTITKEKKEELQDCKIAPLMVAYMLERELVRKTRFLPVAGGTSSVYIEVLHLTQGTCWEQMYTPGGEVRNDDVDQSLIIAARNIVRRATVSADPLASLLEMCHSTQIGGVRMVDILRQNPTEEQAVDICKAAMGLRISSSFSFGGFTFKRTSGSSVKREEEVLTGNLQTLKIRVHEGYEEFTMVGRRATAILRKATRRLIQLIVSGKDEQSIAEAIIVAMVFSQEDCMIKAVRGDLNFVNRANQRLNPMHQLLRHFQKDAKVLFQNWGIEPIDNVMGMIGILPDMTPSTEMSLRGVRVSKMGVDEYSSTERVVVSIDRFLRVRDQRGNILLSPEEVSETQGTEKLTITYSSSLMWEINGPESVLVNTYQWIIRNWETVKIQWSQDPTILYNKMEFEPFQSLVPKAARGQYSGFVRVLFQQMRDVLGTFDTVQIIKLLPFAAAPPEQSRMQFSSLTVNVRGSGMRVVVRGNSPVFNYNKATKRLTVLGKDAGALMEDPDEGTAGVESAVLRGFLILGKEDKRYGPALSINELSNLAKGEKANVLIGQGDVVLVMKRKRDSSILTDSQTATKRIRMAIN

PB1:

MDVNPTLLFLKVPVQNAISTTFPYTGDPPYSHGTGTGYTMDTVNRTHKYSEKGKWTTNTETGAPQLNPIDGPLPEDNEPSGYAQTDCVLEAMAFLEESHPGIFENSCLETMEIVQQTRVDKLTQGRQTYDWTLNRNQPAATALANTIEVFRSNGLTANESGRLIDFLKDVMDSMDKEEMEITTHFQRKRRVRDNMTKKMVTQRTIGKKKQRLNKRSYLIRALTLNTMTKDAERGKLKRRAIATPGMQIRGFVYFVEALARSICEKLEQSGLPVGGNEKKAKLANVVRKMMTNSQDTELSFTITGDNTKWNENQNPRMFLAMITYITRNQPEWFRNVLSIAPIMFSNKMARLGKGYMFESKSMKLRTQVPAEMLANIDLKYFNKSTREKIEKIRPLLIDGTASLSPGMMMGMFNMLSTVLGVSILNLGQKKYTKTTYWWDGLQSSDDFALIVNAPNHEGIQAGVDRFYRTCKLVGINMSKKKSYINRTGTFEFTSFFYRYGFVANFSMELPSFGVSGINESADMSIGVTVIKNNMINNDLGPATAQMALQLFIKDYRYTYRCHRGDTQIQTRRAFELKKLWEQTRSKAGLLVSDGGPNLYNIRNLHIPEVCLKWELMDEDYQGRLCNPMNPFVSHKEIDSVNNAVVMPAHGPAKSMEYDAVATTHSWIPKRNRSILNTSQRGILEDEQMYQKCCNLFEKFFPSSSYRRPVGISSMVEAMVSRARIDARIDFESGRIKKEEFAEIMKICSTIEELRRQK

PB1F2:

MEQEQDTPWTQSTEHINTQKKESGQRTQRLEHPNSIRLMDHCLRTTSRVGMHKRIVYWKQWLSLKNLTQGSLKTRVSKRWKLFSKQEWIN

PA:

MEDFVRQCFNPMIVELAEKAMKEYGEDPKIETNKFASICTHLEVCFMYSDFHFIDERGESTIIESGDPNALLKHRFEIIEGRDRTMAWTVVNSICNTTGVEKPKFLPDLYDYKENRFIEIGVTRREVHIYYLEKANKIKSEKTHIHIFSFTGEEMATKADYTLDEESRARIKTRLFTIRQEMASRGLWDSFRQSERGEETIEERFEITGTMRRLADQSLPPNFSSLENFRAYVDGFEPNGCIEGKLSQMSKEVNARIEPFLRTTPRPLRLPNGPPCSQRSKFLLMDALKLSIEDPSHEGEGIPLYDAIKCMKTFFGWKEPNIIKPHEKGINPNYLLTWKQVLAELQDIENEEKIPRTKNMKKTSQLKWALGENMAPEKVDFEDCKDVNDLKQYDSDEPEPRSLACWIQNEFNKACELTDSSWVELDEIGEDVAPIEHIASMRRNYFTAEVSHCRATEYIMKGVYINTALLNASCAAMDDFQLIPMISKCRTKEGRRKTNLYGFIIKGRSHLRNDTDVVNFVSMEFSLTDPRLEPHKWEKYCVLEIGDMLLRTAVGQVSRPMFLYVRTNGTSKIKMKWGMEMRRCLLQSLQQIESMIEAESSVKEKDLTKEFFENKSETWPIGESPKGVEEGSIGKVCRTLLAKSVFNSLYASPQLEGFSAESRKLLLIVQALRDNLEPGTFDLEGLYEAIEECLINDPWVLLNASWFNSFLTHALR

PA-X:

MEDFVRQCFNPMIVELAEKAMKEYGEDPKIETNKFASICTHLEVCFMYSDFHFIDERGESTIIESGDPNALLKHRFEIIEGRDRTMAWTVVNSICNTTGVEKPKFLPDLYDYKENRFIEIGVTRREVHIYYLEKANKIKSEKTHIHIFSFTGEEMATKADYTLDEESRARIKTRLFTIRQEMASRGLWDSFVSPKEAKKQLKKDLKSQEPCAGLPTKVSHRTSPALKTLEPMWMDSNRTAALRASFLKCQKK

HA:

MNTQILVFALIAIIPTNADKICLGHHAVSNGTKVNTLTERGVEVVNATETVERTNIPRICSKGKKTVDLGQCGLLGTITGPPQCDQFLEFSADLIIERREGSDVCYPGKFVNEEALRQILRESGGIDKEAMGFTYSGIRTNGATSACRRSGSSFYAEMKWLLSNTDNAAFPQMTKSYKNTRKSPALIVWGIHHSVSTAEQTKLYGSGNKLVTVGSSNYQQSFVPSPGARPQVNGLSGRIDFHWLMLNPNDTVTFSFNGAFIAPDRASFLRGKSMGIQSGVQVDANCEGDCYHSGGTIISNLPFQNIDSRAVGKCPRYVKQKSLLLATGMKNVPEIPKGRGLFGAIAGFIENGWEGLIDGWYGFRHQNAQGEGTAADYKSTQSAIDQITGKLNRLIEKTNQQFELIDNEFNEVEKQIGNVINWTRDSITEVWSYNAELLVAMENQHTIDLADSEMDKLYERVKRQLRENAEEDGTGCFEIFHKCDDDCMASIRNNTYDHSKYREEAMQNRIQIDPVKLSSGYKDVILWFSFGASCFILLAIVMGLVFICVKNGNMRCTICI

NP:

MASQGTKRSYEQMETGGERQNATEIRASVGRMVSGIGRFYIQMCTELKLSDNEGRLIQNSITIERMVLSAFDERRNRYLEEHPSAGKDPKKTGGPIYRRRDGKWVRELILYDKEEIRRIWRQANNGEDATAGLTHLMIWHSNLNDATYQRTRALVRTGMDPRMCSLMQGSTLPRRSGAAGAAVKGIGTMVMELIRMIKRGINDRNFWRGENGRRTRIAYERMCNILKGKFQTAAQRAMMDQVRESRNPGNAEIEDLIFLARSALILRGSVAHKSCLPACVYGLAVASGYDFEREGYSLVGIDPFRLLQNSQVFSLIRPNENPAHKSQLVWMACHSAAFEDLRVSSFIRGTRMVPRGQLSTRGVQIASNENMEAMDSNTLELRSRYWAIRTRSGGNTNQQRASAGQVSVQPTFSVQRNLPFERATIMAAFTGNTEGRTSDMRTEIIRMMESARPEDVSFQGRGVFELSDEKATNPIVPSFDMNNEGSYFFGDNAEEYDN

NA:

MNPNQKILCTSATAIIIGAIAVLIGIANLGLNIGLHLKPGCNCSHSQPETTNTSQTIINNYYNETNITNIQMEERTSRNFNNLTKGLCTINSWHIYGKDNAVRIGESSDVLVTREPYVSCDPDECRFYALSQGTTIRGKHSNGTIHDRSQYRALISWPLSSPPTVYNSRVECIGWSSTSCHDGKSRMSICISGPNNNASAVVWYNRRPVAEINTWARNILRTQESECVCHNGVCPVVFTDGSATGPADTRIYYFKEGKILKWESLTGTAKHIEECSCYGERTGITCTCRDNWQGSNRPVIQIDPVAMTHTSQYICSPVLTDNPRPNDPNIGKCNDPYPGNNNNGVKGFSYLDGANTWLGRTISTASRSGYEMLKVPNALTDDRSKPIQGQTIVLNADWSGYSGSFMDYWAEGDCYRACFYVELIRGRPKEDKVWWTSNSIVSMCSSTEFLGQWNWPDGAKIEYFL

M1:

MSLLTEVETYVLSIIPSGPLKAEIAQRLEDVFAGKNADLEALMEWIKTRPILSPLTKGILGFVFTLTVPSERGLQRRRFVQNALNGNGDPNNMDKAVKLYKKLKREMTFHGAKEVALSYSTGALASCMGLIYNRMGTVTAEGALGLVCATCEQIADAQHRSHRQMATTTNPLIRHENRMVLASTTAKAMEQMAGSSEQAAEAMEVASQARQMVQAMRTVGTHPNSSTGLKDDLIENLQAYQNRMGVQLQRFK

M2:

MSLLTEVETLTRTGWECNCSGSSDPLVVAANIIGILHLILWILDRLFFKCIYRRFKYGLKRGPSTEGMPESMREEYRQEQQNAVDVDDGHFVNIELK

NS1:

MDSNTVSSFQVDCFLWHVRKRFADQELGDAPFLDRLRRDQKSLRGRSSTLGLDIRTATREGKHIVERILEEESDEAFKMTIASVPAPRYLTDMTLEEMSRDWLMLIPKQKVTGSLCIRMDQAIVDKNITLKANFSVIFNRLEALILLRAFTDEGAIVGEISPLPSLPGHTDKDVKNAIEILIGGFEWNDNTVRVSETLQRFAWRSSDEDGRPPLSPKXXXXXXXXXXXXX

NS2/NEP:

MDSNTVSSFQDILTRMSKMQLRSSSEDLNGMITQFESLKLYRDSLGEAAMRMGDLHSLQSRNGKWREQLSQKFEEIRWLIEEVRHRLKITENSFEQITFMQALQLLLEVEQEIRTFSFQLI

**2015 H7N9 Avian Consensus Sequences (Theoretical translations of consensus open reading frames, for comparisons in Table 5):**

PB2:

MERIKELRDLMSQSRTREILTKTTVDHMAIIKKYTSGRQEKNPALRMKWMMAMKYPITADKRIMEMIPERNEQGQTLWSKTNDAGSDRVMVSPLAVTWWNRNGPTTSTVHYPKVYKTYFEKVERLKHGTFGPVHFRNQVKIRRRVDINPGHADLSAKEAQDVIMEVVFPNEVGARILTSESQLTITKEKKEELQDCKIAPLMVAYMLERELVRKTRFLPVAGGTSSVYIEVLHLTQGTCWEQMYTPGGEVRNDDVDQSLIIAARNIVRRATVSADPLASLLEMCHSTQIGGVRMVDILRQNPTEEQAVDICKAAMGLRISSSFSFGGFTFKRTSGSSVKREEEVLTGNLQTLKIRVHEGYEEFTMVGRRATAILRKATRRLIQLIVSGKDEQSIAEAIIVAMVFSQEDCMIKAVRGDLNFVNRANQRLNPMHQLLRHFQKDAKVLFQNWGIEPIDNVMGMIGILPDMTPSTEMSLRGVRVSKMGVDEYSSTERVVVSIDRFLRVRDQRGNILLSPEEVSETQGTEKLTITYSSSLMWEINGPESVLVNTYQWIIRNWETVKIQWSQDPTILYNKMEFEPFQSLVPKAARGQYSGFVRVLFQQMRDVLGTFDTVQIIKLLPFAAAPPEQSRMQFSSLTVNVRGSGMRVVVRGNSPVFNYNKATKRLTVLGKDAGALMEDPDEGTAGVESAVLRGFLILGKEDKRYGPALSINELSNLAKGEKANVLIGQGDVVLVMKRKRDSSILTDSQTATKRIRMAIN

PB1:

MDVNPTLLFLKVPVQNAISTTFPYTGDPPYSHGTGTGYTMDTVNRTHKYSEKGKWTTNTETGAPQLNPIDGPLPEDNEPSGYAQTDCVLEAMAFLEESHPGIFENSCLETMEIVQQTRVDKLTQGRQTYDWTLNRNQPAATALANTIEVFRSNGLTANESGRLIDFLKDVMDSMDKEEMEITTHFQRKRRVRDNMTKKMVTQRTIGKKKQRLNKRSYLIRALTLNTMTKDAERGKLKRRAIATPGMQIRGFVYFVEALARSICEKLEQSGLPVGGNEKKAKLANVVRKMMTNSQDTELSFTITGDNTKWNENQNPRMFLAMITYITRNQPEWFRNVLSIAPIMFSNKMARLGKGYMFESKSMKLRTQVPAEMLANIDLKYFNKSTREKIEKIRPLLIDGTASLSPGMMMGMFNMLSTVLGVSILNLGQKKYTKTTYWWDGLQSSDDFALIVNAPNHEGIQAGVDRFYRTCKLVGINMSKKKSYINRTGTFEFTSFFYRYGFVANFSMELPSFGVSGINESADMSXGVTVIKNNMINNDLGPATAQMALQLFIKDYRYTYRCHRGDTQIQTRRAFELKKLWEQTRSKAGLLVSDGGPNLYNIRNLHIPEVCLKWELMDEDYQGRLCNPMNPFVSHKEIDSVNNAVVMPAHGPAKSMEYDAVATTHSWIPKRNRSILNTSQRGILEDEQMYQKCCNLFEKFFPSSSYRRPVGISSMVEAMVSRARIDARIDFESGRIKKEEFAEIMKICSTIEELRRQK

PB1F2:

MEQEQDTPWTQSTEHINTQKKESGQRTQRLEHPNSIQLMDHYLRTMSRVGMHKRIVYWKQWLSLKNLTQGSLKTRVSKRWKLFSKQEWTN

PA:

MEDFVRQCFNPMIVELAEKAMKEYGEDPKIETNKFASICTHLEVCFMYSDFHFIDERGESTIIESGDPNALLKHRFEIIEGRDRTMAWTVVNSICNTTGAEKPKFLPDLYDYKENRFIEIGVTRREVHIYYLEKANKIKSEKTHIHIFSFTGEEMATKADYTLDEESRARIKTRLFTIRQEMASRGLWDSFRQSERGEETIEERFEITGTMRRLADQSLPPNFSSLENFRAYVDGFEPNGCIEGKLSQMSKEVNARIEPFLRTTPRPLRLPNGPPCSQRSKFLLMDALKLSIEDPSHEGEGIPLYDAIKCMKTFFGWKEPNIIKPHEKGINPNYLLTWKQVLAELQDIENEEKIPRTKNMKKTSQLKWALGENMAPEKVDFEDCKDVNDLKQYNSDEPEPRSLACWIQNEFNKACELTDSSWVELDEIGEDVAPIEHIASMRRNYFTAEVSHCRATEYIMKGVYINTALLNASCAAMDDFQLIPMISKCRTKEGRRKTNLYGFIIKGRSHLRNDTDVVNFVSMEFSLTDPRLEPHKWEKYCVLEIGDMLLRTAVGQVSRPMFLYVRTNGTSKIKMKWGMEMRRCLLQSLQQIESMIEAESSVKEKDLTKEFFENKSETWPIGESPKGVEEGSIGKVCRTLLAKSVFNSLYASPQLEGFSAESRKLLLIVQALRDNLEPGTFDLEGLYEAIEECLINDPWVLLNASWFNSFLTHALR

PA-X:

MEDFVRQCFNPMIVELAEKAMKEYGEDPKIETNKFASICTHLEVCFMYSDFHFIDERGESTIIESGDPNALLKHRFEIIEGRDRTMAWTVVNSICNTTGVEKPKFLPDLYDYKENRFIEIGVTRREVHIYYLEKANKIKSEKTHIHIFSFTGEEMATKADYTLDEESRARIKTRLFTIRQEMASRGLWDSFVSPKEAKKQLKKDLKSQEPCAGLPTKVSHRTSPALKTLEPMWMDSNRTAALRASFLRCQKK

HA:

MNTQILVFALIAIIPTNADKICLGHHAVSNGTKVNTLTERGVEVVNATETVERTNIPRICSKGKRTVDLGQCGLLGTITGPPQCDQFLEFSADLIIERREGSDVCYPGKFVNEEALRQILRESGGIDKEAMGFTYNGIRTNGVTSACRRSGSSFYAEMKWLLSNTDNAAFPQMTKSYKNTRKSPAIIVWGIHHSVSTAEQTKLYGSGNKLVTVGSSNYQQSFVPSPGARPQVNGLSGRIDFHWLMLNPNDTVTFSFNGAFIAPDRASFLRGKSMGIQSGVQVDANCEGDCYHSGGTIISNLPFQNIDSRAVGKCPRYVKQRSLLLATGMKNVPEIPKGRGLFGAIAGFIENGWEGLIDGWYGFRHQNAQGEGTAADYKSTQSAIDQITGKLNRLIAKTNQQFELIDNEFNEVEKQIGNVINWTRDSITEVWSYNAELLVAMENQHTIDLADSEMDKLYERVKRQLRENAEEDGTGCFEIFHKCDDDCMASIRNNTYDHRKYREEAMQNRIQIDPVKLSSGYKDVILWFSFGASCFILLAIVMGLVFICVKNGNMRCTICI

NP:

MASQGTKRSYEQMETGGERQNATEIRASVGRMVSGIGRFYIQMCTELKLSDNEGRLIQNSITIERMVLSAFDERRNRYLEEHPSAGKDPKKTGGPIYRRRDGKWVRELILYDKEEIRRIWRQANNGEDATAGLTHLMIWHSNLNDATYQRTRALVRTGMDPRMCSLMQGSTLPRRSGAAGAAVKGIGTMVMELIRMIKRGINDRNFWRGENGRRTRIAYERMCNILKGKFQTAAQRAMMDQVRESRNPGNAEIEDLIFLARSALILRGSVAHKSCLPACVYGLAVASGYDFEREGYSLVGIDPFRLLQNSQVFSLIRPNENPAHKSQLVWMACHSAAFEDLRVSSFIRGTRMVPRGQLSTRGVQIASNENMEAMDSNTLELRSRYWAIRTRSGGNTNQQRASAGQVSVQPTFSVQRNLPFERATIMAAFTGNTEGRTSDMRTEIIRMMESARPEDVSFQGRGVFELSDEKATNPIVPSFDMNNEGSYFFGDNAEEYDN

NA:

MNPNQKILCTSATAIIIGAIAVLIGIANLGLNIGLHLKPGCNCSHSQPETTNTSQTIINNYYNETNITNIQMEERTSRNFNNLTKGLCTINSWHIYGKDNAVRIGESSDVLVTREPYVSCDPDECRFYALSQGTTIRGKHSNGTIHDRSQYRALISWPLSSPPTVYNSRVECIGWSSTSCHDGKSRMSICISGPNNNASAVVWYNRRPVAEINTWARNILRTQESECVCHNGVCPVVFTDGPATGPADTRIYYFKEGKILKWESLTGTAKHIEECSCYGERTGITCTCRDNWQGSNRPVIQIDPVAMTHTSQYICSPVLTDSPRPNDPNIGKCNDPYPGNNNNGVKGFSYLDGANTWLGRTISTASRSGYEMLKVPNALTDDRSKPIQGQTIVLNADWSGYSGSFMDYWAEGDCYRACFYVELIRGRPKEDKVWWTSNSIVSMCSSTEFLGQWNWPDGAKIEYFL

M1:

MSLLTEVETYVLSIIPSGPLKAEIAQRLEDVFAGKNADLEALMEWIKTRPILSPLTKGILGFVFTLTVPSERGLQRRRFVQNALNGNGDPNNMDKAVKLYKKLKREMTFHGAKEVALSYSTGALASCMGLIYNRMGTVTAEGALGLVCATCEQIADAQHRSHRQMATTTNPLIRHENRMVLASTTAKAMEQMAGSSEQAAEAMEVASQARQMVQAMRTVGTHPNSSTGLKDDLIENLQAYQNRMGVQLQRFK

M2:

MSLLTEVETLTRTGWECNCSGSSDPLVVAANIIGILHLILWILDRLFFKCIYRRFKYGLKRGPSTEGMPESMREEYRQEQQNAVDVDDGHFVNIELK

NS1:

MDSNTVSSFQVDCFLWHVRKRFADQEMGDAPFLDRLRRDQKSLRGRSSTLGLDIRTATREGKHIVERILEEESDEAFKMSIASVPAPRYLTDMTLEEMSRDWLMLIPKQKITGSLCIRMDQAIVDKNITLKANFSVIFNRLEALILLRAFTEEGAIVGEISPLPSLPGHTDKDVKNAIEILIGGFEWNDNTVRVSETLQRFAWRSSDEDGRSPLSTKXXXXXXXXXXXXX

NS2/NEP:

MDSNTVSSFQDILTRMSKMQLRSSSEDLNGMITQFESLKLYRDSLGEAAMRMGDLHSLQSRNGKWREQLSQKFEEIRWLIEEVRHRLKITENSFEQITFMQALQLLLEVEQEIRTFSFQLI
